# Adipose tissue aging: mechanisms and therapeutic implications

**DOI:** 10.1038/s41419-022-04752-6

**Published:** 2022-04-04

**Authors:** Min-Yi Ou, Hao Zhang, Poh-Ching Tan, Shuang-Bai Zhou, Qing-Feng Li

**Affiliations:** Department of Plastic & Reconstructive Surgery, Shanghai Ninth People’s Hospital, Shanghai Jiao Tong University School of Medicine, 200011 Shanghai, China

**Keywords:** Senescence, Endocrine system and metabolic diseases

## Abstract

Adipose tissue, which is the crucial energy reservoir and endocrine organ for the maintenance of systemic glucose, lipid, and energy homeostasis, undergoes significant changes during aging. These changes cause physiological declines and age-related disease in the elderly population. Here, we review the age-related changes in adipose tissue at multiple levels and highlight the underlying mechanisms regulating the aging process. We also discuss the pathogenic pathways of age-related fat dysfunctions and their systemic negative consequences, such as dyslipidemia, chronic general inflammation, insulin resistance, and type 2 diabetes (T2D). Age-related changes in adipose tissue involve redistribution of deposits and composition, in parallel with the functional decline of adipocyte progenitors and accumulation of senescent cells. Multiple pathogenic pathways induce defective adipogenesis, inflammation, aberrant adipocytokine production, and insulin resistance, leading to adipose tissue dysfunction. Changes in gene expression and extracellular signaling molecules regulate the aging process of adipose tissue through various pathways. In addition, adipose tissue aging impacts other organs that are infiltrated by lipids, which leads to systemic inflammation, metabolic system disruption, and aging process acceleration. Moreover, studies have indicated that adipose aging is an early onset event in aging and a potential target to extend lifespan. Together, we suggest that adipose tissue plays a key role in the aging process and is a therapeutic target for the treatment of age-related disease, which deserves further study to advance relevant knowledge.

## Facts

- Impact of age on range adipose tissue cellular and molecular composition, which can be detected at middle age.
- The cause of age-related change in adipose tissue is complicated, ranging from external factors and internal senescence.
- Age-related adipose tissue alterations that accelerate the systemic aging process are promising therapeutic targets to prevent age-related disease.

## Introduction

Intensified aging of the population is generally accompanied by increased age-related diseases that impair the quality of life of the elderly. Aging is characterized by progressive physiological declines and is the greatest cause of human pathologies and death worldwide [1, 2]. However, deleterious processes interact with extraordinary complexity within and between organs, and the underlying mechanisms are still poorly understood. One of the most vulnerable tissues in aging is adipose tissue, with alterations in several biological and physiological processes that in turn impact the overall well-being of the organism. In a recent study, researchers found that an age-related immune response was first detected in white adipose depots [3]. As adipose tissue is an attractive stem cell pool for regenerative transplantation, age-modifying treatments that eliminate age-associated dysfunction in adipose-derived stem cells can improve the efficiency of stem cell therapy [4]. As the largest energy storage and endocrine organ, adipose tissue plays a significant role in energy and metabolism homeostasis. The dysfunctional adipose tissue in aging promotes low-grade chronic inflammation, insulin resistance, and lipid infiltration in the elderly [5–7]. Since adipose tissue is a potential target for aging intervention, it is of significance to take a whole picture of the aging process of adipose tissue.

Adipose tissues have been traditionally divided into white adipose tissue (WAT) and brown adipose tissue (BAT) [8]. WAT is the predominant lipid storage and is involved in multiple immunoendocrine responses. BAT that enhances energy consumption is mainly located in the interscapular space of mice but can be found in the interscapular, supraclavicular, suprarenal, and para-aortic space among others of humans, according to the age of the subject [9]. In the course of cold exposure, BAT is in charge of maintaining body temperature by nonshivering thermogenesis [10]. Beige fat cells are derived from WAT depots with a brown fat-like morphology and function [11]. Adipose tissue can also be categorized according to the specific depots, with subcutaneous fat (SAT) and visceral fat (VAT) being a large proportion of WAT and being investigated in many previous studies. Adipose tissue undergoes dramatic changes in various aspects during aging [12]. Fat is redistributed in aging with decreased SAT and increased intra-abdominal visceral depots. A decrease in brown and beige fat leads to thermal dysregulation and energy imbalance. In addition to adipocytes and adipose progenitor cells, other nonadipocyte cells, such as macrophages, fibroblasts, and lymphocytes, are also indispensable components of the stromal vascular fraction (SVF) and contribute to the hallmarks of aging [13–15]. Due to the decreased inflammatory and coagulant-related gene expression in resident stromal cells of adipose tissue, the elderly population is significantly more susceptible to inflammatory stress [16]. Elevated cellular senescence and the related senescence-associated secretory phenotype (SASP) are significant features of aging, which are proposed to play an essential role in the age-related functional decline of adipose tissue [17]. Extensive research has investigated the underlying biomechanical and biological mechanisms of adipose tissue aging, especially excess adiposity.

As a large and dynamic endocrine, immune, and regenerative organ, adipose tissue plays a major role in health via releasing factors that regulate diverse processes, such as appetite control, glucose metabolism, insulin sensitivity, inflammation activity, and tissue repair [18–20]. According to the previous expression profile of active genes in adipose tissue, approximately 20–30% of genes expressed in white adipose tissue produce secreted proteins [21]. Increasing adipocyte-secreted endocrine factors affecting adjacent or remote tissues and organs have been identified, with well-recognized leptin and adiponectin selectively expressed in adipocytes [22]. Due to the dysregulation in endocrine factors, the ability of adipose tissue to buffer excess nutrients is reduced with advancing age, which probably let older people be more prone to obesity [23]. Moreover, adipose tissue becomes dysfunctional with a dysregulated secretome, including proinflammatory cytokines and hormones, which is correlated with several known age-related disorders. Adipose tissue-derived substances or stimuli contribute to the widespread presence of chronic inflammation in aging directly or indirectly. Obesity, a common disease generally characterized by abnormal adipose tissue, is supposed to be a state of accelerated aging [24]. Inflammation and oxidative stress seem to be important mediators of the complicated association between obesity and the aging process. Interestingly, preclinical and clinical studies show that strategies targeting adipose tissue aging have been shown to mitigate age-associated physical dysfunction and extend health span. In this review, we discuss how aging impacts fat tissue function and in turn leads to age-related disease, with the related cell biological and molecular mechanisms. Additionally, we discuss the attractive role of aging adipose tissue in aging therapy.

## Features of adipose tissue aging

### Redistribution of adipose tissue

**Fig. 1:**
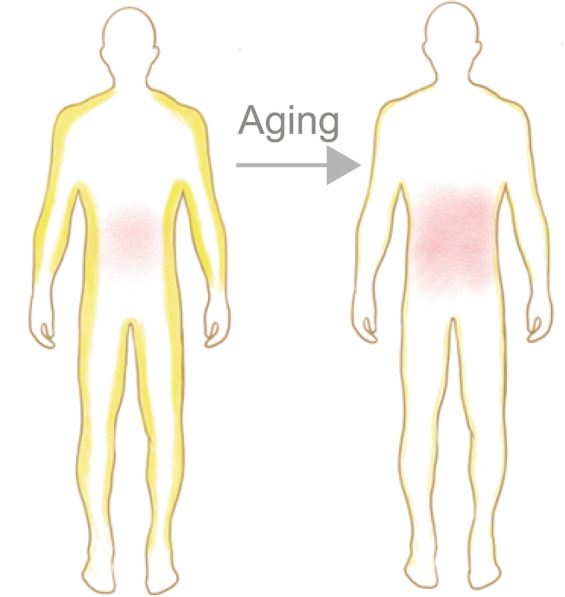
The age-related redistribution of adipose tissue. There is a redistribution of WAT mass with advancing age, displayed by increased visceral fat and reduced subcutaneous fat. Yellow represents subcutaneous fat, and red represents visceral fat.

In the process of aging, adipose tissue undergoes dramatic changes in mass and biodistribution. Total fat mass accumulation is common in both healthy and unhealthy elderly individuals and can occur as early as middle age [25]. However, a decline in fat mass can be found in extremely old stages and may be a sign of deteriorated health [25]. Another age-related change in body composition is fat redistribution, which is featured by a preferential increase in visceral fat, with a decrease in lower body subcutaneous fat [12] (Fig. 1). Subcutaneous and visceral adipose depots are very different in terms of their effects on metabolism. In general, SAT is considered beneficial for metabolism, whereas VAT is thought to be harmful. The age-related redistribution of adipose tissue in favor of visceral depots impacts systematic healthy aging. Thus, fat redistribution during aging is correlated with an increased risk of metabolic abnormalities, particularly insulin resistance accompanied by an increased risk of cardiovascular disease and diabetes [25, 26].

The underlying mechanisms of subcutaneous peripheral fat loss during aging are not fully appreciated. It has been suggested that peripheral fat loss in aging is partially due to defects in adipogenesis in SAT, which are related to overactivated inflammation [27]. In addition, the telomere basal length in subcutaneous fat is shorter than that in visceral fat, suggesting that SAT is more vulnerable to age-related detriment [28]. Considering the beneficial effects of SAT on systemic metabolism, metabolic dysfunction in aging probably originates from SAT deficiency.

### Reduced brown and beige fat

The age-related alterations vary in different depots of adipose tissue, with a significant reduction in BAT and beige fat that are critical thermogenic cells for maintaining body temperature by nonshivering thermogenesis. In general, brown and beige adipocytes enhance their activity to increase energy expenditure, which is thought to resist adipose tissue dysfunction and the development of obesity [29].

The decline of brown and beige fat occurs in the aging process, which possibly aggravates thermal dysregulation and energy imbalance in elderly individuals [30, 31]. The decline in BATs is first detected in interscapular depots during aging. However, deeper depots, particularly perivascular and kidney depots, are lost at a later stage. The decreased BAT is consistent with the fact that older humans are hard to maintain body temperature. Researchers found that *UCP1* and *β3AR* are associated with age-related reductions in BAT activity [32]. Increased winged helix factor forkhead box protein A3 (FOXA3) expression in aging adipose tissue is also involved in the reduction of BAT [33]. Increasing BAT by RGS14 knockout or surgical BAT transplantation results in healthful aging with enhanced longevity and metabolism [34]. It is considerable to take BAT as a therapeutic target for the health of elderly people.

### Functional decline of adipose progenitor and stem cells

Adipose progenitor and stem cells (APSCs) are the essential stem cell pool in the stromal vascular fraction (SVF) of adipose tissue and give birth to mature adipocytes. The differentiation and proliferation potential of APSCs ensures the renewal, expansion, and functional plasticity of adipose tissue. However, the proliferation and differentiation capacity of APSCs gradually declines with increasing age [4, 35]. In addition to the dramatically slower growth rate of preadipocytes in older individuals, the differentiation of preadipocytes is significantly compromised compared to that in younger individuals. APSCs obtained from older donors have less osteogenic potential than those obtained from young donors, which suggests that aging APSCs might only have limited suitability for regenerative medicine [36]. The decline in APSC proliferation is reported to begin at age 30 and is most obvious at age 50 years. APSC dysfunction impairs the plasticity of adipose tissue, which may be an underlying mechanism of insulin resistance in elderly individuals [37]. Furthermore, because preadipocytes are less able to differentiate and properly store lipids, aging adipose tissue exposes other tissues and organs to even greater amounts of lipotoxic free fatty acids [38]. This lipotoxicity is recognized as a critical mechanism of metabolic syndrome that seriously impacts the quality of life of elderly people. Several therapeutics targeting APSCs have been developed and have achieved some interesting results. Blocking activin A by JAK inhibition was demonstrated to be a useful strategy for improving senescent APSCs, with restored lipid accumulation and expression of key adipogenic markers [39]. Fat grafting, which applies the multidirectional differentiation and reproductive activity of adipose-derived progenitors, has been utilized for over 100 years [40]. Whether and how aging impacts fat grafting in elderly individuals? How can the stemness of APSCs be enhanced for fat grafting in old patients? These questions require more research to answer.

### Accumulation of senescent cell

**Fig. 2:**
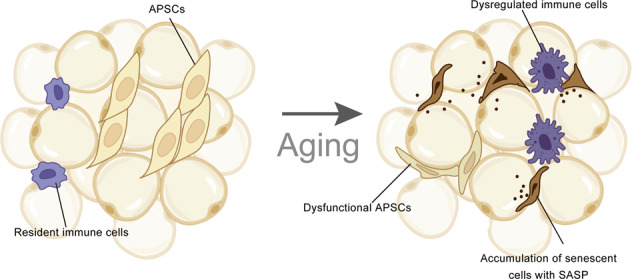
Age-related alteration in adipose tissue with dysregulated immune cells, preadipocytes and senescent cells. In young WAT, APSCs actively proliferate and differentiate to adipocytes. Resident immune cells keep in a relatively stable state. With advancing aging, APSCs gradually lose their developmental potential, leading to low adipogenesis. Aberrant immune cells and senescent cells accumulation drive the aging process of adipose. This figure was created in part with modified BioRender templates.

Cellular senescence is a state characterized by cell cycle arrest and is related to a decline in the regenerative potential and function of various tissues, which drive the systematic aging process [41]. Adipose tissue is a site of massive senescent cell accumulation during aging [42, 43] (Fig. 2). Senescent cells accumulate in aging fat induced by a combination of replicative, cytokine-induced, and metabolic stresses [44]. Although cellular senescence is suggested to be a defensive mechanism preventing tumorigenesis, its occurrence in adipose tissue causes multiple dysfunctions, including defective adipogenesis, inflammation, aberrant adipocytokine production, and insulin resistance. These aging cells secrete the SASP consisting of cytokines, chemokines, proteases, and growth factors, which is considered to be an aging signal [39, 44] (Fig. 2). The declined stemness and adipogenesis of aged APSCs may also be a result of the accumulation of senescent cells [39]. A study found that human senescent adipocyte progenitors inhibit adipogenesis of surrounding nonsenescent progenitors via the paracrine pathway [39]. Only 20% of adipose progenitors accumulate lipids when cocultured with senescent cells, compared to more than 50% of progenitors cocultured with nonsenescent cells. These effects may be related to activin A, interleukin-6 (IL-6), TNF-α, interferon-γ (IFN-γ), and/or the SASP components of senescent adipose progenitor cells and/or other senescent cell types. When the accumulation of senescent cells is too much to clean, the response of immune cells may be disturbed by chemokines released by senescent cells.

### Changes in immune cells

Abnormal activation of immune cells is a hallmark of aging and is first detected in WAT depots at middle age (Fig. 2). WAT harbors a complex combination of immune cells, including lymphocytes, macrophages, and eosinophils. Various endogenous substances or stress-inducing agents, such as hypoxia and excess nutritional element-related fatty acids, byproducts of cell death, and endoplasmic reticulum (ER) stressors, may trigger sterile inflammation of adipose tissue to varying degrees. However, we still lack knowledge about the context of immune cells in aging adipose tissue. The increased expression of VSIG4 (CRIg/Z39Ig), a macrophage-associated protein that regulates both innate and adaptive immunity, correlates with age and physiological frailty in mice, which indicates the alteration of macrophages in adipose tissue aging [45]. Adipose tissue macrophages regulate the age-related decline in adipocyte lipolysis in mice by repressing the bioavailability of noradrenaline, which can be rescued by the deletion of catecholamine degradation genes [7]. Aged ILC2s are compromised to a proinflammatory and senescence-like state with dysregulated IL-33 expression, ultimately leading to a cold vulnerability in old mice [14]. Adipose tissue eosinophils undergo major age-related changes in distribution and function with eosinophil-derived IL-4 deficiency [46]. Fat-resident regulatory T cells increase in aged adipose tissue, which is supposed to regulate adipose tissue insulin sensitivity [47]. Fat-resident B cell dysfunction with high TNF-α levels has been reported in a previous study, which may lead to an impaired influenza vaccine-specific response in elderly individuals [48]. The immunosuppressive network in aging is supposed to prevent excessive inflammatory responses, but at the same time, they repress the immune system [49]. Very few studies have investigated the role of the immunosuppressive network in adipose aging. It is controversial whether the changes in immune cells within aged adipose tissue are a cause or consequence of adipose tissue dysfunction, which needs further investigation.

## Mechanisms of adipose tissue dysfunction during aging

### Decline in brown and beige fat function

Resident BAT in adults can be found mostly in the cervical-supraclavicular region and smaller depots located in the axillary, mediastinal, paravertebral, epicardial, and abdominal areas [50], which is related to poor temperature regulation in elderly individuals. Several mechanisms are associated with BAT decline with advancing age. Mitochondrial function is impaired in adipose tissue in age, which may be due to the accumulation of mitochondrial DNA mutations, as well as a reduction in oxidative phosphorylation and the expression of the uncoupled activity of protein-1 (UCP-1), a thermogenesis-related mitochondrial protein in brown fat cells [51]. Since the sympathetic nervous system mediates the activation of BAT at cold temperatures, low sympathetic activity in older individuals may contribute to poor BAT activity [52]. Increased levels of proinflammatory cytokines in the aging process repress BAT thermogenic capacity by suppressing UCP-1 gene expression [53, 54]. Glucocorticoids inhibit adrenergic-stimulated UCP-1 expression, which may contribute to a decline in BAT activity [55, 56]. The intervention of hormone levels could be a strategy to preserve, supported by the finding that inhibition of circulating orexigenic hormone Ghrelin by gene editing or antagonist in mice increased thermogenic capacity in brown adipose tissues [57].

A reduction in beige adipocyte formation is also detected in aging adipose tissue. The age-related reduction in SIRT1, which drives beige adipocyte generation from WAT, maybe one of the key mechanisms in the loss of beige adipose tissue [58]. Together, enhancing the function of BAT is a promising strategy to mitigate age‐associated thermogenic impairment.

### Functional defects in APSCs

**Fig. 3:**
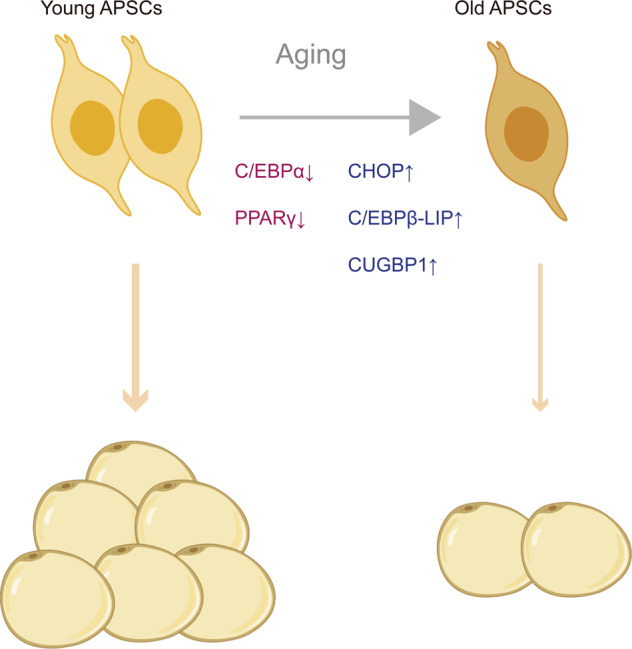
Mechanisms of impaired adipogenesis with aging. In the aging process, the expression of pro-adipogenic genes, such as C/EBPα and PPARγ, declines in adipose progenitor and stem cells (APSCs), accompanied by increased levels of antiadipogenic factors, including C/EBPβ-LIP, CHOP, and CUGBP1. As a result, old APSCs show a decline in proliferation and differentiation, which leads to low adipogenesis in the elderly. This figure was created in part with modified BioRender templates.

A mechanistic understanding of APSC dysfunction with age could help to prevent age-related adipose disorders. Impaired preadipocyte differentiation is linked to altered levels of adipogenic factors (Fig. 3). C/EBP family members and PPARγ play vital roles in the differentiation program by regulating the transcription of adipogenic genes [59]. Previous studies have demonstrated that the expression of C/EBPα, a pivotal regulator of preadipocyte differentiation initiation and adipocyte maintenance, is decreased in fat tissue in older humans compared to younger humans [60]. Age-related decline in PPARγ expression in adipose tissue also contributes to impaired adipogenesis during aging [61, 62]. Moreover, impaired differentiation capacity during aging is a result, in part, of increased levels of antiadipogenic factors. CCAAT/enhancer-binding protein beta liver-inhibitory protein (C/EBPβ-LIP) and CCAAT/enhancer-binding protein homologous protein (CHOP) are upregulated with aging, which dampens the differentiation of adipocytes [63, 64]. CUG triplet repeat-binding protein-1 (CUGBP1) abundance and activity increase in aging adipose tissue, which may be related to stress responses, thereby resisting adipogenesis via enhancing C/EBPβ-LIP translation [63]. Thus, inhibition of CUGBP expression in the preadipocytes of elderly persons may help to improve adipogenesis.

In addition to transcription factors, microRNAs (miRNAs) and short (17–20 nts) noncoding RNAs are also involved in preadipocyte dysfunction during the aging process by regulating transcription and mRNA translation in adipogenic pathways. Age-associated differentiation defects of preadipocytes with aging are found to be correlated with miR-143, which promotes adipocyte differentiation via the ERK5-PPARγ pathway [62, 65]. Together, the key genes whose expression level correlated with age in preadipocytes are potential targets to rejuvenate preadipocytes in elderly individuals.

### Accumulation of senescent cells with the SASP

Senescent cell accumulation can be induced by various endogenous and exogenous stresses, including DNA damage, telomere shortening, oncogenic mutations (e.g., Ras, Myc, and B-Raf), and environmental stresses (e.g., protein aggregation and unfolded proteins) [66]. Consistent with the notion that cellular senescence is an important mechanism for preventing cancer progression, the P53 and pRB tumor pathways are central regulators of senescent cell accumulation [67]. Inhibition of P53 has been shown to induce senescent cells to reenter the cell cycle [68, 69]. Chronic inflammation with continued upregulation of proinflammatory mediators (e.g., TNF-α, IL-1β, 6, COX-2, iNOS) may promote cell senescence, which in turn becomes a source of proinflammatory secretion [70]. SASP factors derived from senescent cells in adipose tissue contribute to proinflammatory factors (cytokines, chemokines, and microRNAs), TNF receptors, nonprotein soluble factors (nitric oxide), growth factors (EGF, VEGF, and NGF), and extracellular matrix macromolecules (fibronectin, collagens, and laminin) in the microenvironment [43, 71]. The role of the SASP may not be limited to impacting tissue structure and function directly or indirectly. SASP factors such as IL-6, IL-8, GROα, and IGFBP-7 participate in an autocrine feedback loop to reinforce growth arrest in senescent cells for tumor suppression [72]. In addition, the SASP might recruit an infiltrating immune response to clear senescent cells [73]. Several molecular mechanisms have been demonstrated to participate in SASP regulation, which could be potential targets for age-related therapy. NF-κB and C/EBPβ, which are activated in senescent cells, mediate the upregulation of SASP components at the mRNA level [74]. Inhibition of the Janus kinase/signal transducer and activator of transcription (JAK/STAT) pathway, which plays a significant role in adipose tissue development and function and regulates the SASP, can partially inhibit SASP secretion [75]. Since the roles of cell senescence and the SASP in aging are complicated, future studies need to precisely elucidate the deleterious effects of the SASP and cell senescence.

### Low-level inflammation in adipose tissue

Chronic inflammation characterized by continued proinflammatory factor secretion at levels higher than baseline contributes to general aging and age-related diseases [76]. Fat tissue, especially preadipocytes, has been suggested to be a major source of inflammatory cytokines during aging. A previous study reported that adipose tissue in old mice expresses higher levels of proinflammatory cytokines, including IL-1, IL-6, TNF-α, and the hallmark lipid inflammatory mediator cyclooxygenase 2 (COX-2), and lower levels of anti-inflammatory PPAR-gamma than those of young mice [76]. Another aging study found that genes of cytokine-mediated inflammatory pathways, including Ccl8, show significantly upregulated expression in GAT at 18 months [3]. Proinflammatory cytokine release from preadipocytes under TNF-α exposure drives adjacent cells into a proinflammatory state, in parallel with promoting endothelial cell-monocyte adhesion and macrophage infiltration [77]. Moreover, TNF-α release from undifferentiated preadipocytes with high C/EBP and CHOP expression impaired adipogenesis, which aggravated age-related dysfunction in adipose tissue [64]. Age-related activation of inflammatory cytokines and chemokine expression in adipose tissue vary among different depots, e.g., an age-related increase in the IL-6 response is higher in subcutaneous fat than in visceral fat, which is consistent with disproportional defects in subcutaneous fat [78]. Although age-related changes in the fat tissue inflammatory environment are similar to those in obesity, the inflammatory expansion and activation of macrophages in aging seem less impressive than those in obesity [79]. The response of macrophages to shift into a proinflammatory state by chemokines and cytokines generally declines with aging [80].

Several mechanisms have been shown to induce inflammation with aging. Dysregulated autophagy activity in aging adipose tissue promotes age-related high elevated endoplasmic reticulum (ER) stress and inflammation, which may be linked to the accumulation of autophagy substrates LC3-II and p62 [81]. The ER stress response in aging adipose tissue promotes age-associated inflammation, which can be attenuated by chemical chaperones [6]. Since dysregulated adipokines from adipose tissue can contribute to chronic low-grade inflammation in obesity, adipokines may also play a role in age-related inflammation [23].

### Metabolic dysfunctions of adipose tissue, particularly insulin resistance

Several age-related changes in adipose tissue have been supposed to be the endogenic reason for metabolic dysfunction. Adipocyte hypertrophy with inefficient nutrient transport and poor cell signaling, which is a common feature of aging, may lead to metabolic defects and decreased energy expenditure [82]. Age-related changes in the adipose tissue extracellular matrix, such as decreased periostin and collagen VI, may lead to metabolic defects by negatively affecting nutrient and energy homeostasis [83, 84]. Ablation of the gene encoding periostin in mice leads to age-related metabolic dysfunction with a low adaptation of adipose tissue to adrenergic stimulation and high-fat diet feeding, as well as lipid metabolism in adipose tissue [83]. Cellular senescence in adipose tissue probably participates in metabolic dysfunction, which is supported by the finding that inhibition of p53 activity in adipose tissue markedly improved insulin resistance [85]. Consistently, inhibition of senescent cells or their products in adipose tissue has been shown to improve metabolism in old mice [39]. Accumulating evidence indicates that age-related alterations in adipose tissue contribute to insulin resistance in the elderly population. Age-related defects in the insulin signaling cascade, such as a reduction in insulin-stimulated tyrosine phosphorylation, are more severe in adipose tissue than in either the liver or muscle, which suggests that adipose tissue may be an origin of insulin resistance during the aging process [86]. Lipid redistribution and chronic inflammation derived from aging adipose tissue induce metabolic perturbation, including insulin resistance, impaired glucose tolerance, and diabetes. High levels of proinflammatory cytokines, such as IL-1 family members, in dysfunctional adipose tissue may directly disturb the insulin signaling pathway [87, 88]. In addition, age-related alteration of immune cells, such as T cell accumulation, maybe one of the causes of insulin resistance [47].

## Aging changes in adipose tissue impact the whole body (secretion/metabolic effect)

### Age-related changes in adipose tissue impact metabolism in nonadipose tissue

With the widespread communication between adipose tissue and nonadipose tissue, altered adipose tissues impact the metabolism of other organs and tissue via several pathways. Both the thermogenesis activity and total mass of brown and beige adipose tissue decline with advancing age, which contributes to decreased energy consumption in the elderly population. Impaired cold exposure-stimulated thermogenesis of BAT also causes age-associated cold sensitivity. Since BAT regulates whole-body glucose and energy homeostasis via consuming fuels such as glucose and lipids, a decline in BAT in aging can cause a preference for metabolic disease [89]. In addition, BAT dysfunction disturbs the metabolism of other tissues with altered secretion, which mediates the conversation between different organs. As 12,13-diHOME acts as a paracrine signal to promote skeletal muscle fatty acid uptake and oxidation, decreased 12,13-diHOME from BAT in old mice may counteract energy consumption under conditions such as cold exposure or exercise [90, 91]. It has been reported that the accumulation of perimuscular adipose tissue, which is ectopic fat deposition surrounding atrophied muscle, promotes age-related muscle atrophy by increasing proteolysis and in muscle [92]. The breakdown and release of lipids in aging due to lack of lipid-storing adipocytes and decreased lipolysis in impaired WAT lead to lipotoxicity on other tissue [5]. For instance, ectopic lipid accumulation in the liver may accelerate the development of nonalcoholic fatty liver disease during aging [93]. In addition, age-related redistribution of white adipose tissue, especially with excessive adipose tissue mass in visceral, intermuscular, and intramuscular depots, exacerbates system-wide metabolic dysfunction. Increased visceral adipose mass, which can impair the liver with free fatty acids and proinflammatory factors, may be particularly involved in the pathogenesis of insulin resistance and type 2 diabetes [94]. Together, metabolic defects both locally and systematically caused by adipose tissue ultimately make older people more susceptible to various metabolic diseases.

### Abnormal adipose-derived hormones in the aging have broad effects

Adipose tissue is an important endocrine organ that releases adipokines, such as leptin, adiponectin, and resistin, that are signals to target other tissues and organs [95]. The abnormal adipokine levels released from either adipocytes or adipose tissue-infiltrated macrophages in aging serve to the chronic inflammatory environment and insulin resistance, which could be a risk factor for cardiovascular disease in elderly individuals. Leptin, which is a circulating hormone/cytokine mainly secreted from subcutaneous WAT, participates in feeding and energy homeostasis [96]. Impaired leptin-mediated regulation with leptin resistance, which probably results from high plasma leptin levels and declined WAT, induces abnormal hypothalamus-related activity and elevated obesity and serum leptin with age [97, 98]. The correlation of leptin levels with liver diseases, such as cirrhosis and fibrosis, supports that leptin may mediate the pernicious influence of abnormal adipose tissue on the liver [99–101]. Adiponectin, an adipocyte-derived sensitizer of insulin signaling, and its downstream factors have been implicated in insulin signaling, which is vulnerable to aging [98]. Adiponectin reduces the triglyceride content in skeletal muscle by increasing molecules involved in fatty acid translocation to protect insulin-stimulated phosphatidylinositol (PI) 3-kinase activation and glucose metabolism, which is required for proper insulin signaling [102]. Dysregulation of adiponectin in older individuals is related to aging-associated chronic diseases, which might be a potential target for aging therapy [103]. Adipocyte-derived resistin antagonizes insulin signaling, in parallel with decreasing glucose intake in adipocytes, muscle cells, and other tissues. Resistin induces increased permeability and superoxide anion production in coronary artery endothelial cells, which may contribute to vascular lesion formation and subsequent vascular disease [104]. In brief, dysregulated adipokines due to age-related alterations may spread the aging signal and accelerate the aging process.

### Adipose-derived proinflammatory cytokines contribute to systemic inflammation

In the process of aging, high circulating levels of proinflammatory cytokines derived from adipose tissue are regarded as major contributors to systemic, chronic low-grade inflammation. Previous research reported that 30% of circulating IL-6, a well-recognized inflammatory factor, is produced by WAT, with visceral WAT expressing higher IL-6 levels than subcutaneous WAT [78, 105, 106]. High levels of IL-6 and MCP-1 from preadipocytes responding to ER stress in aging are inflammatory cytokines that have broad effects on elderly individuals [6]. As adipose macrophage content has been shown to positively correlate with aging, macrophage-derived NLRP3 inflammasomes may cooperate with activated T cells to induce the development of inflammation in adipose tissue and the liver [107, 108]. Since TNF-α is an essential proinflammatory cytokine that drives the inflammatory process, whether dysfunctional adipose tissue induces systemic inflammation by secreting TNF-α in aging may need further investigation [109, 110]. Through the ERK/ETS1/interleukin-27Ra (IL27Ra) pathway, age-related TNF-α causes functional decline and myeloid bias of hematopoietic stem cells, which is related to myeloproliferative disease and immunosenescence [111]. Harmful products produced by dysregulated adipose tissue, such as lipids and free fatty acids, are also driving factors of inflammation [7]. Increased systemic free fatty acids, especially saturated fatty acids, promote the binding of monocytes to endothelial cells and proatherogenic cell surface antigen expression, which is a risk factor for atherosclerosis [112–114].

### Alterations in aging adipose tissue can lead to systematic changes by circulating miRNAs

MiRNAs are small noncoding RNAs with 19–22 nucleotides that play critical roles in regulating mRNA metabolism. Circulating miRNAs are present in the extracellular environment, of which a large proportion are wrapped in exosomes [115]. In addition to being recognized as biomarkers [116], miRNAs in systemic circulation could regulate the gene expression and function of distal cells by mediating paracrine and endocrine communication between different tissues. Adipose tissue has been demonstrated to be a crucial source of circulating miRNA, which may be involved in the communication between adipose tissue and other tissues. For instance, mouse miR-99b derived from BAT regulates FGF21 expression in the liver [117]. Decreased miRNA biogenesis due to the downregulation of Dicer and miRNA processing occurs at the level of the whole organism, including adipose tissue [117]. Interestingly, the beneficial effects of caloric restriction on the aging process rely on appropriate miRNAs from fat tissue, which may include circulating miRNAs [118]. To apply adipose-derived miRNAs for aging therapy, we still need more future studies to identify detailed miRNAs released from adipose tissue.

### Obesity is a risk factor for age-related diseases

It has long been established that obesity is one of the major risk factors for a range of age-related diseases, including diabetes, cancer, and cardiovascular disease [119]. There are many causes of obesity, including genetic mutation, physical inactivity, and malnutrition, with the common result of abnormal accumulation of fat tissue. Obesity-related adipocyte hyperplasia in visceral white adipose tissue, which occurs more often in males than in females, may drive the age-associated redistribution of adipose tissue [120]. Obesity has been shown to accelerate the aging process in the liver, with nuclear mitochondrial genes involved in phosphorylation and electron transport [121]. In obesity, the excess presence of ROS due to fat accumulation increased Gfi1 expression in hematopoietic stem cells, which is associated with age-related hematological disorders [122–124]. Increased retinol-binding protein-4 (RBP4) in obesity downregulates phosphatidylinositol-3-OH kinase (PI(3)K) signaling in muscle and promotes gluconeogenic enzyme phosphoenolpyruvate carboxykinase expression in the liver through a retinol-dependent mechanism, which induces insulin resistance [125]. For cardiovascular disease, studies have revealed a significant association between obesity and elevated blood pressure, with obese individuals being 3.5 times more likely to develop hypertension [126]. Obesity-induced adipose tissue inflammation may be a driver of cancer risk, and certain adipokines (e.g., CCL2, VEGF, IL-6, and IL-8) act as chemotherapy inducers that enhance tumor cell migration and support metastasis. A recent study also found that obesity represses the infiltration and function of CD8+ T cells in the murine tumor microenvironment, which accelerates tumor growth [127]. Since obesity and the aging process share many pathogeneses and phenotypes, including impaired mitochondrial function, abnormal immunity, elevated systemic inflammation, and insulin resistance, interventions for obesity could slow down the aging process.

## Adipose tissue as a therapeutic target in aging

### White adipose depots show early onset of aging

Recently, a large-scale targeted proteomic analysis of a diverse panel of young versus old murine tissues revealed a significant aging effect on white adipose tissue, with alterations in lipid metabolism, central carbon metabolism, electron transport chain complexes, and inflammation [128]. Another bulk RNA sequencing of 17 organs at 10 ages across the lifespan of mice suggested that WAT is an early onset of aging [3]. In WAT, a significant increase in differentially expressed genes in older mice compared to 3-month-old adults was detected at mid-age, which is earlier than that in other organs. Researchers analyzed the expression of genes involved in the age-associated immune response and found significant activation of cytokine-mediated inflammation in adipose tissue. Widespread activation of immune cells with accumulation of T cells and B cells, which is generally related to the aging onset, was first detected in WAT during middle age [3]. By correlating plasma protein age trajectories with their corresponding gene expression trajectories in each organ, researchers also found that WAT is the source of several age-plasma proteins, which may accelerate the aging process throughout the body. In addition, impaired plasticity of subcutaneous WAT is already evident in middle-aged mice, which may be the early reason for insulin resistance [129].

### Systemic therapies extend lifespan by improving fat aging

Strategies against the harmful influence of the aging process have recently been developed, some of which come into work by targeting adipose tissue. With the combination of dasatinib-a and quercetin-tyrosine kinase inhibitors (D + Q), Senolytics could alleviate physical dysfunction, including maximal walking speed, hanging endurance, and grip strength, in parallel with extending lifespan and reducing mortality hazard in 24–27 months mice (equivalent to 75–90-year-old human) [130]. In an open-label phase 1 pilot study of Senolytics, old patients with diabetic kidney disease treated with Senolytics showed a reduction in adipose tissue senescent cell burden and key SASP factors within 11 days [131]. In addition, a recent study demonstrated that aging-related alterations in the systemic environment partially originate in white adipose depots [46]. Thus, intervention for adipose tissue aging may serve to repress age-related diseases and extend lifespan. In particular, the specific reduction in senescent cell accumulation and proinflammatory cytokine secretion in adipose tissue was the same as that in Senolysis-treated humans and mice, which supports that adipose tissue aging is a potential target for aging therapy [131]. Metformin is a well-known drug that has been used to treat T2D for >60 years and has been a popular star in anti-aging research in recent decades [132]. One of the mechanisms of Metformin in aging therapy is that Metformin improves PPAR and SREBP signaling, mitochondrial fatty acid oxidation, and collagen trimerization in adipose tissue [133]. Recent work reported that the low-dose PPARγ agonist thiazolidinedione (TZD) might be a novel pharmacological intervention to counteract aging and extend lifespan. Experiments in old mice showed that eWAT had the highest degree of gene expression changes in response to TZD treatment, specifically in inflammatory responses. Mice treated with TZD displayed improved age-dependent adipose tissue loss and reduced inflammation and fibrosis in aging WAT, contributing to the maintenance of adipose tissue homeostasis [134]. Heterochronic parabiosis studies with a fusion of the circulatory systems of young mice and aged mice suggest that blood factors from younger donors can rejuvenate physiological function in old mice partially through a diminished expression of cyclin-dependent kinase inhibitor (CDKi) genes p16 (Cdkn2a) and p21 (Cdkn1a/Cip1) in VAT [135]. To reach the beneficial effect of CR more efficiently, pharmacological approaches, named CR mimetics (CRMs), that mimic the role of CR in health have been introduced. Consistently, CRMs have profound effects on adipose tissue. Metformin, a popular CRM, prevents abnormal white adipocyte accumulation by increasing FGF21 expression [136].

### Adipose-specific interventions affect longevity

Caloric restriction (CR) is a common strategy to prevent abnormal fat accumulation by chronic reduction of total calorie intake without malnutrition [137, 138]. The expression of genes associated with proliferator-activated receptor γ (PPARγ)-mediated adipogenesis lipid metabolism was downregulated with age but preserved by CR in WAT [139]. In addition to preventing obesity-related pathologies through weight loss, CR has also been broadly demonstrated to extend health span in most living organisms [140, 141]. CR treatment for 24 months had a beneficial effect in nonobese humans, which increased vigor, reduced mood disturbance, and improved sleep quality [142]. An increasing underlying mechanism of CR against aging has been identified. As CR suppressed a substantial subset of the age-associated changes in WAT [139], adipose tissue might act as an important mediator of the beneficial effects of CR, directly or indirectly. CR prevents the age-related accumulation of adipose tissue, which causes a series of damage on the adjacent or distant organs. Previous research suggested that sirtuin 1 (SIRT1) is the key molecule that mediates the effect of CR on lifespan by inhibiting lipid accumulation and promoting lipolysis in adipocytes [143]. Moreover, CR with nutrient deprivation activates appropriate autophagy levels to remove dysfunctional organelles, proteins, and aggregates from the cytoplasm by regulating the expression of key genes, such as AMP-activated protein kinase (AMPK) [144]. The general reduction in mammalian target of rapamycin (mTOR) activity in aging can also be rescued to some degree by CR [139]. Interestingly, surgical removal of VAT in rats offered approximately 20% of the effect of CR on longevity with improving insulin action [145].

Although highly controversial, gene therapy is still a promising treatment for various diseases and has moved from a vision to a clinical reality. Finding the key gene target is fundamental for gene therapy against aging. Intriguingly, editing several adipose-related genes was shown to extend the lifespan to varying degrees. The expression of *Nrip1* in visceral white adipose tissue (WAT) increases with aging, which might be associated with VAT expansion in aging. Nrip1 deletion in mice increases autophagy activity in periovarian white adipose tissue and reduces cellular senescence and proinflammatory cytokines in WAT, ultimately extending the health span [146]. Deleting Toll-like receptors in mice can alleviate inflammation at old age by reducing inflammation-related processes, including ER stress and senescence, which is a promising antiaging therapy [147].

Together, adipose tissue interventions through lifestyle, drugs, and gene editing can result in better health, suggesting that adipose tissue is a worthy target for treatment against aging.

## Summary

**Fig. 4:**
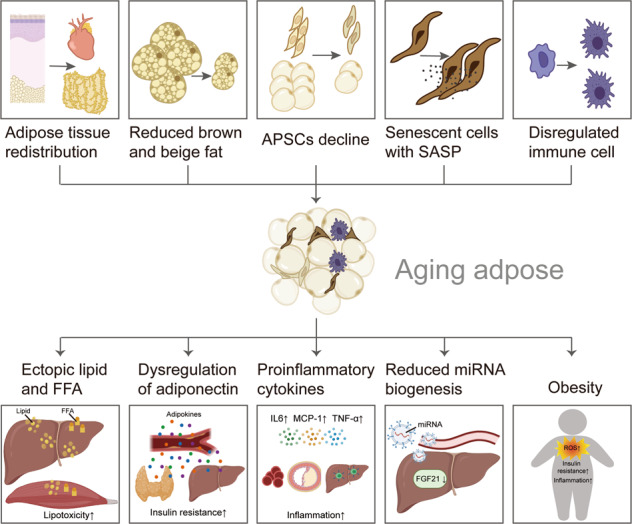
The role of adipose tissue in the aging process. In aging individuals, the adipose tissue can be characterized by tissue redistribution, reduced brown and beige fat, declined APSCs, senescent cell accumulation with SASP, and dysregulated immune cells. Aging adipose impacts the elderly with ectopic lipid and FFA, dysregulated adiponectin, increased proinflammatory cytokines, reduced miRNA synthesis, and high ROS activity. This figure was created in part with modified BioRender templates.

As the lifespan of human beings has been extended greatly, it is important to find ways to reach healthful aging with physical and mental vigor. A large number of studies have emphasized the important role of adipose tissue in aging. Adipose tissue plays a crucial role in nutrient sensing, energy storage, and endocrine and immunological activity. Age-related adipose tissue alterations, including abnormal redistribution, decreased progenitor pool, accumulated senescent cells, and activated inflammation, accelerate the aging process in the local environment, which can drive systemic adverse health outcomes with advancing age (Fig. 4). Slowing down the aging process in adipose tissue is thought to prevent age-related disease. In this review, we provide mechanistic insights into the aging progression of adipose tissue with a cascade of molecular and cellular changes, as well as the underlying mechanism. Notably, some molecules derived from adipose tissue, such as free fatty acids, extracellular lipids, and SASP, promote aging at the organismal level. Nevertheless, there remains some unresolved problem: What makes adipose tissue act at the onset of aging? Which cell type in adipose tissue is the origin of aging? Whether all adipocytes undergo aging synchronously? Is the role of the immune response in adipose aging a protector? Thanks to the rapid evolution of single-cell technologies, it has been possible to investigate the aging process of adipose tissue within several cell types. As adipose aging intervention has the potential to protect against systematic aging and age-related disease, more research is required to unveil the detailed mechanisms underlying fat aging and to provide a theoretical basis for antiaging therapy.

## Supplementary Information

Supplementary Information

## Data Availability

The data used to support the findings of this study are available from the corresponding author upon request.
