# Supplementary material for: Adipose tissue aging: mechanisms and therapeutic implications
Source: Cell Death Dis. 2022 Apr 4;13(4):300. doi: 10.1038/s41419-022-04752-6 (PMC8980023; doi:10.1038/s41419-022-04752-6)
Supplement: Supplementary file 1 — Supplementary Information [file 41419_2022_4752_MOESM1_ESM.docx]

**Tittle:** **Adipose tissue aging: Mechanisms and** **therapeutic implications**

**Authors:** Min-Yi Ou^1,#^, Hao Zhang^1,#^, Poh-Ching Tan^1^, Shuang-Bai Zhou^1^, Qing-Feng Li^1^

1 Department of Plastic & Reconstructive Surgery, Shanghai Ninth People’s Hospital, Shanghai Jiao Tong University School of Medicine, Shanghai 200011, China

**Correspondence:**

Shuang-Bai Zhou. and Qing-Feng Li,

Department of Plastic & Reconstructive Surgery,

Shanghai Ninth People's Hospital, Shanghai Jiao Tong University, 639 Zhizhaoju Road,

Shanghai, P.R. China, 200011

Email**:** [shuangbaizhou@yahoo.com](mailto:shuangbaizhou@yahoo.com) (S. B. Zhou), qf_li@yahoo.com (Q. F. Li)

# These authors contributed equally to this study.

**Abstract**

Adipose tissue, which is the crucial energy reservoir and endocrine organ for the maintenance of systemic glucose, lipid and energy homeostasis, undergoes significant changes during aging. These changes cause physiological declines and age-related disease in the elderly population. Here, we review the age-related changes in adipose tissue at multiple levels and highlight the underlying mechanisms regulating the aging process. We also discuss the pathogenic pathways of age-related fat dysfunctions and their systemic negative consequences, such as dyslipidemia, chronic general inflammation, insulin resistance, and type 2 diabetes (T2D). Age-related changes in adipose tissue involve redistribution of deposits and composition, in parallel with functional decline of adipocyte progenitors and accumulation of senescent cells. Multiple pathogenic pathways induce defective adipogenesis, inflammation, aberrant adipocytokine production and insulin resistance, leading to adipose tissue dysfunction. Changes in gene expression and extracellular signaling molecules regulate the aging process of adipose tissue through various pathways. In addition, adipose tissue aging impacts other organs that are infiltrated by lipids, which leads to systemic inflammation, metabolic system disruption, and aging process acceleration. Moreover, studies have indicated that adipose aging is an early onset event in aging and a potential target to extend lifespan. Together, we suggest that adipose tissue plays a key role in the aging process and is a therapeutic target for the treatment of age-related disease, which deserves further study to advance relevant knowledge.

**Facts**

Impact of age on range adipose tissue cellular and molecular composition, which can be detected at middle age.

The cause of age-related change in adipose tissue is complicated, ranging from external factors and internal senescence.

Age-related adipose tissue alterations that accelerate the systemic aging process, are promising therapeutic target to prevent age-related disease.

**Introduction**

Intensified aging of the population is generally accompanied by increased age-related diseases that impair the quality of life of the elderly. Aging is characterized by progressive physiological declines and is the greatest cause of human pathologies and death worldwide^1, 2^. However, deleterious processes interact with extraordinary complexity within and between organs, and the underlying mechanisms are still poorly understood. One of the most vulnerable tissues in aging is adipose tissue, with alterations in several biological and physiological processes that in turn impact the overall well-being of the organism. In a recent study, researchers found that an age-related immune response was first detected in white adipose depots^3^. As adipose tissue is an attractive stem cell pool for regenerative transplantation, age-modifying treatments that eliminate age-associated dysfunction in adipose-derived stem cells can improve the efficiency of stem cell therapy^4^. As the largest energy storage and endocrine organ, adipose tissue plays a significant role in energy and metabolism homeostasis. The dysfunctional adipose tissue in aging promotes low grade chronic inflammation, insulin resistance, and lipid infiltration in the elderly^5, 6, 7^. Since adipose tissue is a potential target for aging intervention, it is of significance to take a whole picture of the aging process of adipose tissue.

Adiposes tissue have been traditionally divided into white adipose tissue (WAT) and brown adipose tissue (BAT)^8^. WAT is the predominant lipid storage and is involved in multiple immunoendocrine responses. BAT that enhances energy consumption, mainly locates in interscapular space of mice, but can be found in the interscapular, supraclavicular, suprarenal and para-aortic space among others of human, according to the age of the subject^9^. In the course of cold exposure, BAT is in charge of maintaining body temperature by nonshivering thermogenesis^10^. Beige fat cells are derived from WAT depots with a brown fat-like morphology and function ^11^. Adipose tissue can also be categorized according to the specific depots, with subcutaneous fat (SAT) and visceral fat (VAT) being a large proportion of WAT and being investigated in many previous studies. Adipose tissue undergoes dramatic changes in various aspects during aging^12^. Fat is redistributed in aging with decreased SAT and increased intra-abdominal visceral depots. A decrease in brown and beige fat leads to thermal dysregulation and energy imbalance. In addition to adipocytes and adipose progenitor cells, other nonadipocyte cells, such as macrophages, fibroblasts and lymphocytes, are also indispensable components of the stromal vascular fraction (SVF) and contribute to the hallmarks of aging^13, 14, 15^. Due to the decreased inflammatory and coagulant-related gene expression in resident stromal cells of adipose tissue, the elderly population is significantly more susceptible to inflammatory stress^16^. Elevated cellular senescence and the related senescence-associated secretory phenotype (SASP) are significant features of aging, which are proposed to play an essential role in the age-related functional decline of adipose tissue^17^. Extensive research has investigated the underlying biomechanical and biological mechanisms of adipose tissue aging, especially excess adiposity.

As a large and dynamic endocrine, immune, and regenerative organ, adipose tissue plays a major role in health via releasing factors that regulate diverse processes, such as appetite control, glucose metabolism, insulin sensitivity, inflammation activity and tissue repair^18, 19, 20^. According to the previous expression profile of active genes in adipose tissue, approximately 20–30% of genes expressed in white adipose tissue produce secreted proteins^21^. Increasing adipocyte-secreted endocrine factors affecting adjacent or remote tissues and organs have been identified, with well-recognized leptin and adiponectin selectively expressed in adipocytes^22^. Due to the dysregulation in endocrine factors, the ability of adipose tissue to buffer excess nutrients is reduced with advancing age, which probably let older people to be more prone to obesity^23^. Moreover, adipose tissue becomes dysfunctional with a dysregulated secretome, including proinflammatory cytokines and hormones, which is correlated with several known age-related disorders. Adipose tissue-derived substances or stimuli contribute to the widespread presence of chronic inflammation in aging directly or indirectly. Obesity, a common disease generally characterized by abnormal adipose tissue, is supposed to be a state of accelerated aging^24^. Inflammation and oxidative stress seem to be important mediators of the complicated association between obesity and the aging process. Interestingly, preclinical and clinical studies show that strategies targeting adipose tissue aging have been shown to mitigate age-associated physical dysfunction and extend healthspan. In this review, we discuss how aging impacts fat tissue function and in turn leads to age-related disease, with the related cell biological and molecular mechanisms. Additionally, we discuss the attractive role of aging adipose tissue in aging therapy.

**1. Features of Adipose Tissue Aging**

**(1) Redistribution of adipose tissue**

In the process of aging, adipose tissue undergoes dramatic changes in mass and biodistribution. Total fat mass accumulation is common in both healthy and unhealthy elderly individuals and can occur as early as middle age^25^. However, a decline in fat mass can be found in extremely old stages and may be a sign of deteriorated health^25^. Another age-related change in body composition is fat redistribution, which is featured by a preferential increase in visceral fat, with a decrease in lower body subcutaneous fat^12^ (Figure 1). Subcutaneous and visceral adipose depots are very different in terms of their effects on metabolism. In general, SAT is considered beneficial for metabolism, whereas VAT is thought to be harmful. The age-related redistribution of adipose tissue in favor of visceral depots impacts systematic healthy aging. Thus, fat redistribution during aging is correlated with an increased risk of metabolic abnormalities, particularly insulin resistance accompanied by an increased risk of cardiovascular disease and diabetes^25, 26^.

The underlying mechanisms of subcutaneous peripheral fat loss during aging are not fully appreciated. It has been suggested that peripheral fat loss in aging is partially due to defects in adipogenesis in SAT, which are related to overactivated inflammation ^27^. In addition, the telomere basal length in subcutaneous fat is shorter than that in visceral fat, suggesting that SAT is more vulnerable to age-related detriment^28^. Considering the beneficial effects of SAT on systemic metabolism, metabolic dysfunction in aging probably originates from SAT deficiency.

**(2) Reduced brown and beige fat**

The age-related alterations vary in different depot of adipose tissue, with significant reduction in BAT and beige fat that are critical thermogenic cells for maintaining body temperature by nonshivering thermogenesis. In general, brown and beige adipocytes enhance their activity to increase energy expenditure, which is thought to resist adipose tissue dysfunction and the development of obesity^29^.

The decline of brown and beige fat occurs in the aging process, which possibly aggravates thermal dysregulation and energy imbalance in elderly individuals^30^ ^31^. The decline in BATs is first detected in interscapular depots during aging. However, deeper depots, particularly perivascular and kidney depots, are lost at a later stage. The decreased BAT is consistent with the fact that older humans are hard to maintain body temperature. Researchers found that *UCP1* and *β3AR* are associated with age-related reductions in BAT activity^32^. Increased winged helix factor forkhead box protein A3 (FOXA3) expression in aging adipose tissue is also involved in the reduction of BAT^33^. Increasing BAT by RGS14 knockout or surgical BAT transplantation results in healthful aging with enhanced longevity and metabolism^34^. It is considerable to take BAT as a therapeutic target for the health of elderly people.

**(3) Functional decline of adipose progenitor and stem cells**

Adipose progenitor and stem cells (APSCs) are the essential stem cell pool in the stromal vascular fraction (SVF) of adipose tissue and give birth to mature adipocytes. The differentiation and proliferation potential of APSCs ensures the renewal, expansion and functional plasticity of adipose tissue. However, the proliferation and differentiation capacity of APSCs gradually declines with increasing age^4, 35^. In addition to the dramatically slower growth rate of preadipocytes in older individuals, the differentiation of preadipocytes is significantly compromised compared to that in younger individuals. APSCs obtained from older donors have less osteogenic potential than those obtained from young donors, which suggests that ageing APSCs might only have limited suitability for regenerative medicine^36^. The decline in APSC proliferation is reported to begin at age 30 and is most obvious at age 50. APSC dysfunction impairs the plasticity of adipose tissue, which may be an underlying mechanism of insulin resistance in elderly individuals^37^. Furthermore, because preadipocytes are less able to differentiate and properly store lipids, aging adipose tissue exposes other tissues and organs to even greater amounts of lipotoxic free fatty acids^38^. This lipotoxicity is recognized as a critical mechanism of metabolic syndrome that seriously impacts the quality of life of elderly people. Several therapeutics targeting APSCs have been developed and have achieved some interesting results. Blocking activin A by JAK inhibition was demonstrated to be a useful strategy for improving senescent APSCs, with restored lipid accumulation and expression of key adipogenic markers^39^. Fat grafting, which applies the multidirectional differentiation and reproductive activity of adipose-derived progenitors, has been utilized for over 100 years^40^. Whether and how aging impacts fat grafting in elderly individuals? How can the stemness of APSCs be enhanced for fat grafting in old patients? These questions require more research to answer.

**(4) Accumulation of senescent cell**

Cellular senescence is a state characterized by cell cycle arrest and is related to a decline in the regenerative potential and function of various tissues, which drive the systematic aging process^41^. Adipose tissue is a site of massive senescent cell accumulation during aging^42, 43^ (Figure 2). Senescent cells accumulate in ageing fat induced by a combination of replicative, cytokine-induced, and metabolic stresses^44^. Although cellular senescence is suggested to be a defensive mechanism preventing tumorigenesis, its occurrence in adipose tissue causes multiple dysfunctions, including defective adipogenesis, inflammation, aberrant adipocytokine production and insulin resistance. These aging cells secrete the SASP consisting of cytokines, chemokines, proteases, and growth factors, which is considered to be an aging signal^39, 44^ (Figure 2). The declined stemness and adipogenesis of aged APSCs may also be a result of the accumulation of senescent cells^39^. A study found that human senescent adipocyte progenitors inhibit adipogenesis of surrounding nonsenescent progenitors via the paracrine pathway^39^. Only 20% of adipose progenitors accumulate lipids when cocultured with senescent cells, compared to more than 50% of progenitors cocultured with nonsenescent cells. These effects may be related to activin A, interleukin-6 (IL-6), TNF-α, interferon-γ (IFN-γ), and/or the SASP components of senescent adipose progenitor cells and/or other senescent cell types. When the accumulation of senescent cells is too much to clean, the response of immune cells may be disturbed by chemokines released by senescent cells.

**(5) Changes in immune cells**

Abnormal activation of immune cells is a hallmark of aging and is first detected in WAT depots at middle age (Figure 2). WAT harbors a complex combination of immune cells, including lymphocytes, macrophages, and eosinophils. Various endogenous substances or stress-inducing agents, such as hypoxia and excess nutritional element-related fatty acids, byproducts of cell death, and endoplasmic reticulum (ER) stressors, may trigger sterile inflammation of adipose tissue to varying degrees. However, we still lack knowledge about the context of immune cells in ageing adipose tissue. The increased expression of VSIG4 (CRIg/Z39Ig), a macrophage-associated protein that regulates both innate and adaptive immunity, correlates with age and physiological frailty in mice, which indicates the alteration of macrophages in adipose tissue aging^45^. Adipose tissue macrophages regulate the age-related decline in adipocyte lipolysis in mice by repressing the bioavailability of noradrenaline, which can be rescued by deletion of catecholamine degradation genes^7^. Aged ILC2s are compromised to a proinflammatory and senescence-like state with dysregulated IL-33 expression, ultimately leading to cold vulnerability in old mice^14^. Adipose tissue eosinophils undergo major age-related changes in distribution and function with eosinophil-derived IL-4 deficiency^46^. Fat-resident regulatory T cells increase in aged adipose tissue, which is supposed to regulate adipose tissue insulin sensitivity^47^. Fat-resident B cell dysfunction with high TNF-α levels has been reported in a previous study, which may lead to an impaired influenza vaccine-specific response in elderly individuals^48^. The immunosuppressive network in aging is supposed to prevent excessive inflammatory responses, but at the same time, they repress the immune system^49^. Very few studies have investigated the role of the immunosuppressive network in adipose aging. It is controversial whether the changes in immune cells within aged adipose tissue are a cause or consequence of adipose tissue dysfunction, which needs further investigation.

**2. Mechanisms of Adipose Tissue Dysfunction During Aging**

**(1) Decline in brown and beige fat function**

Resident BAT in adults can be found mostly in the cervical-supraclavicular region and smaller depots located in the axillary, mediastinal, paravertebral, epicardial, and abdominal areas ^50^, which is related to poor temperature regulation in elderly individuals. Several mechanisms are associated with BAT decline with advancing age. Mitochondrial function is impaired in adipose tissue in age, which may be due to the accumulation of mitochondrial DNA mutations, as well as a reduction in oxidative phosphorylation and the expression of the uncoupled activity of protein-1 (UCP-1), a thermogenesis-related mitochondrial protein in brown fat cells^51^. Since the sympathetic nervous system mediates the activation of BAT at cold temperatures, low sympathetic activity in older individuals may contribute to poor BAT activity ^52^. Increased levels of proinflammatory cytokines in the aging process repress BAT thermogenic capacity through suppressing UCP-1 gene expression^53, 54^. Glucocorticoids inhibit adrenergic-stimulated UCP-1 expression, which may contribute to a decline in BAT activity^55, 56^. Intervention of hormone levels could be a strategy to preserve, supported by the finding that inhibition of circulating orexigenic hormone Ghrelin by gene editing or antagonist in mice increased thermogenic capacity in brown adipose tissues^57^.

A reduction in beige adipocyte formation is also detected in aging adipose tissue. The age-related reduction in SIRT1, which drives beige adipocyte generation from WAT, may be one of the key mechanisms in the loss of beige adipose tissue^58^. Together, enhancing the function of BAT is a promising strategy to mitigate age‐associated thermogenic impairment.

**(2) Functional defects in APSCs**

A mechanistic understanding of APSC dysfunction with age could help to prevent age-related adipose disorders. Impaired preadipocyte differentiation is linked to altered levels of adipogenic factors (Figure 3). C/EBP family members and PPARγ play vital roles in the differentiation program by regulating the transcription of adipogenic genes^59^. Previous studies have demonstrated that the expression of C/EBPα, a pivotal regulator of preadipocyte differentiation initiation and adipocyte maintenance, is decreased in fat tissue in older humans compared to younger humans^60^. Age-related decline in PPARγ expression in adipose tissue also contributes to impaired adipogenesis during aging^61, 62^. Moreover, impaired differentiation capacity during aging is a result, in part, of increased levels of antiadipogenic factors. CCAAT/enhancer-binding protein beta liver-inhibitory protein (C/EBPβ-LIP) and CCAAT/enhancer-binding protein homologous protein (CHOP) are upregulated with aging, which dampens the differentiation of adipocytes^63, 64^. CUG triplet repeat-binding protein-1 (CUGBP1) abundance and activity increase in aging adipose tissue, which may be related to stress responses, thereby resisting adipogenesis via enhancing C/EBPβ-LIP translation ^63^. Thus, inhibition of CUGBP expression in the preadipocytes of elderly persons may help to improve adipogenesis.

In addition to transcription factors, microRNAs (miRNAs) and short (17–20 nt) noncoding RNAs are also involved in preadipocyte dysfunction during the aging process by regulating transcription and mRNA translation in adipogenic pathways. Age-associated differentiation defects of preadipocytes with aging are found to be correlated with miR-143, which promotes adipocyte differentiation via the ERK5-PPARγ pathway ^62, 65^. Together, the key genes whose expression level correlated with age in preadipocytes are potential targets to rejuvenate preadipocytes in elderly individuals.

**(3) Accumulation of senescent cells with the SASP**

Senescent cell accumulation can be induced by various endogenous and exogenous stresses, including DNA damage, telomere shortening, oncogenic mutations (e.g., Ras, Myc, and B-Raf), and environmental stresses (e.g., protein aggregation and unfolded proteins)^66^. Consistent with the notion that cellular senescence is an important mechanism for preventing cancer progression, the P53 and pRB tumor pathways are central regulators of senescent cell accumulation^67^. Inhibition of P53 has been shown to induce senescent cells to reenter the cell cycle^68, 69^. Chronic inflammation with continued upregulation of proinflammatory mediators (e.g., TNF-α, IL-1β, 6, COX-2, iNOS) may promote cell senescence, which in turn becomes a source of proinflammatory secretion^70^. SASP factors derived from senescent cells in adipose tissue contribute to proinflammatory factors (cytokines, chemokines, and microRNAs), TNF receptors, nonprotein soluble factors (nitric oxide), growth factors (EGF, VEGF, and NGF), and extracellular matrix macromolecules (fibronectin, collagens, and laminin) in the microenvironment^43, 71^. The role of the SASP may not be limited to impacting tissue structure and function directly or indirectly. SASP factors such as IL-6, IL-8, GROα and IGFBP-7 participate in an autocrine feedback loop to reinforce growth arrest in senescent cells for tumor suppression^72^. In addition, the SASP might recruit an infiltrating immune response to clear senescent cells^73^. Several molecular mechanisms have been demonstrated to participate in SASP regulation, which could be potential targets for age-related therapy. NF-κB and C/EBPβ, which are activated in senescent cells, mediate the upregulation of SASP components at the mRNA level^74^. Inhibition of the Janus kinase/signal transducer and activator of transcription (JAK/STAT) pathway, which plays a significant role in adipose tissue development and function and regulates the SASP, can partially inhibit SASP secretion^75^. Since the roles of cell senescence and the SASP in aging are complicated, future studies need to precisely elucidate the deleterious effects of the SASP and cell senescence.

**(4) Low-level inflammation in adipose tissue**

Chronic inflammation characterized by continued proinflammatory factor secretion at levels higher than baseline contributes to general aging and age-related diseases^76^. Fat tissue, especially preadipocytes, has been suggested to be a major source of inflammatory cytokines during aging. A previous study reported that adipose tissue in old mice expresses higher levels of proinflammatory cytokines, including IL-1, IL-6, TNF-α and the hallmark lipid inflammatory mediator cyclooxygenase 2 (COX-2), and lower levels of anti-inflammatory PPAR-gamma than those of young mice ^76^. Another aging study found that genes of cytokine-mediated inflammatory pathways, including Ccl8, show significantly upregulated expression in GAT at 18 months^3^. Proinflammatory cytokine release from preadipocytes under TNF-α exposure drives adjacent cells into a proinflammatory state, in parallel with promoting endothelial cell-monocyte adhesion and macrophage infiltration ^77^. Moreover, TNF-α release from undifferentiated preadipocytes with high C/EBP and CHOP expression impaired adipogenesis, which aggravated age-related dysfunction in adipose tissue^64^. Age-related activation of inflammatory cytokines and chemokine expression in adipose tissue vary among different depots, e.g., an age-related increase in the IL-6 response is higher in subcutaneous fat than in visceral fat, which is consistent with disproportional defects in subcutaneous fat^78^. Although age-related changes in the fat tissue inflammatory environment are similar to those in obesity, the inflammatory expansion and activation of macrophages in aging seems less impressive than those in obesity^79^. The response of macrophages to shift into a proinflammatory state by chemokines and cytokines generally declines with aging^80^.

Several mechanisms have been shown to induce inflammation with aging. Dysregulated autophagy activity in aging adipose tissue promotes age-related high elevated endoplasmic reticulum (ER) stress and inflammation, which may be linked to the accumulation of autophagy substrates LC3-II and p62 ^81^. The ER stress response in aging adipose tissue promotes age-associated inflammation, which can be attenuated by chemical chaperones^6^. Since dysregulated adipokines from adipose tissue can contribute to chronic low-grade inflammation in obesity, adipokines may also play a role in age-related inflammation^23^.

**(5) Metabolic dysfunctions of adipose tissue, particularly insulin resistance**

Several age-related changes in adipose tissue have been supposed to be the endogenic reason for metabolic dysfunction. Adipocyte hypertrophy with inefficient nutrient transport and poor cell signaling, which is a common feature of aging, may lead to metabolic defects and decreased energy expenditure^82^. Age-related changes in the adipose tissue extracellular matrix, such as decreased periostin and collagen VI, may lead to metabolic defects by negatively affecting nutrient and energy homeostasis^83, 84^. Ablation of the gene encoding periostin in mice leads to age-related metabolic dysfunction with low adaptation of adipose tissue to adrenergic stimulation and high-fat diet feeding, as well as lipid metabolism in adipose tissue^83^. Cellular senescence in adipose tissue probably participates in metabolic dysfunction, which is supported by the finding that inhibition of p53 activity in adipose tissue markedly improved insulin resistance^85^. Consistently, inhibition of senescent cells or their products in adipose tissue has been shown to improve metabolism in old mice^39^. Accumulating evidence indicates that age-related alterations in adipose tissue contribute to insulin resistance in the elderly population. Age-related defects in the insulin signaling cascade, such as a reduction in insulin-stimulated tyrosine phosphorylation, are more severe in adipose tissue than in either the liver or muscle, which suggests that adipose tissue may be an origin of insulin resistance during the aging process^86^. Lipid redistribution and chronic inflammation derived from aging adipose tissue induce metabolic perturbation, including insulin resistance, impaired glucose tolerance, and diabetes. High levels of proinflammatory cytokines, such as IL-1 family members, in dysfunctional adipose tissue may directly disturb the insulin signaling pathway^87, 88^. In addition, age-related alteration of immune cells, such as T cell accumulation, may be one of the causes of insulin resistance^47^.

**3. Aging changes in adipose tissue impact the whole body (secretion/metabolic effect)**

**(1) Age-related changes in adipose tissue impact metabolism in nonadipose tissue**

As the widespread communication between adipose tissue and nonadipose tissue, altered adipose tissues impact the metabolism of other organs and tissue via several pathways. Both the thermogenesis activity and total mass of brown and beige adipose tissue decline with advancing age, which contributes to decreased energy consumption in the elderly population. Impaired cold exposure-stimulated thermogenesis of BAT also causes age-associated cold sensitivity. Since BAT regulates whole-body glucose and energy homeostasis via consuming fuels such as glucose and lipids, a decline in BAT in aging can cause a preference for metabolic disease^89^. In addition, BAT dysfunction disturbs the metabolism of other tissues with altered secretion, which mediates the conversation between different organs. As 12,13-diHOME acts as a paracrine signal to promote skeletal muscle fatty acid uptake and oxidation, decreased 12,13-diHOME from BAT in old mice may counteract energy consumption under conditions such as cold exposure or exercise ^90, 91^. It has been reported that the accumulation of perimuscular adipose tissue, which is ectopic fat deposition surrounding atrophied muscle, promotes age-related muscle atrophy by increasing proteolysis and in muscle^92^. The breakdown and release of lipids in aging due to lack of lipid-storing adipocytes and decreased lipolysis in impaired WAT, which leads to lipotoxicity on other tissue^5^. For instance, ectopic lipid accumulation in the liver may accelerate the development of nonalcoholic fatty liver disease during aging^93^. In addition, age-related redistribution of white adipose tissue, especially with excessive adipose tissue mass in visceral, intermuscular, and intramuscular depots, exacerbates system-wide metabolic dysfunction. Increased visceral adipose mass, which can impair the liver with free fatty acids and proinflammatory factors, may be particularly involved in the pathogenesis of insulin resistance and type 2 diabetes^94^. Together, metabolic defects both locally and systematically caused by adipose tissue ultimately make older people more susceptible to various metabolic diseases.

**(2) Abnormal adipose-derived hormones in aging have broad effects.**

Adipose tissue is an important endocrine organ that releases adipokines, such as leptin, adiponectin and resistin, that are signals to target other tissues and organs^95^. The abnormal adipokine levels released from either adipocytes or adipose tissue-infiltrated macrophages in aging serves to the chronic inflammatory environment and insulin resistance, that could be a risk factor for cardiovascular disease in elderly individuals. Leptin, which is a circulating hormone/cytokine mainly secreted from subcutaneous WAT, participates in feeding and energy homeostasis^96^. Impaired leptin-mediated regulation with leptin resistance, which probably results from high plasma leptin levels and declined WAT, induces abnormal hypothalamus-related activity and elevated obesity and serum leptin with age^97, 98^. The correlation of leptin levels with liver diseases, such as cirrhosis and fibrosis, supports that leptin may mediate the pernicious influence of abnormal adipose tissue on the liver^99, 100, 101^. Adiponectin, an adipocyte-derived sensitizer of insulin signaling, and its downstream factors have been implicated in insulin signaling, which is vulnerable to aging^98^. Adiponectin reduces the triglyceride content in skeletal muscle by increasing molecules involved in fatty acid translocation to protect insulin-stimulated phosphatidylinositol (PI) 3-kinase activation and glucose metabolism, which is required for proper insulin signaling^102^. Dysregulation of adiponectin in older individuals is related to aging-associated chronic diseases, which might be a potential target for aging therapy^103^. Adipocyte-derived resistin antagonizes insulin signaling, in parallel with decreasing glucose intake in adipocytes, muscle cells, and other tissues. Resistin induces increased permeability and superoxide anion production in coronary artery endothelial cells, which may contribute to vascular lesion formation and subsequent vascular disease^104^. In brief, dysregulated adipokines due to age-related alterations may spread the aging signal and accelerate the aging process.

**(3) Adipose-derived proinflammatory cytokines contribute to systemic inflammation**

In the process of aging, high circulating levels of proinflammatory cytokines derived from adipose tissue are regarded as major contributors to systemic, chronic low-grade inflammation. Previous research reported that 30% of circulating IL-6, a well-recognized inflammatory factor, is produced by WAT, with visceral WAT expressing higher IL-6 levels than subcutaneous WAT^78, 105, 106^. High levels of IL-6 and MCP-1 from preadipocytes responding to ER stress in aging are inflammatory cytokines that have broad effects on elderly individuals^6^. As adipose macrophage content has been shown to positively correlate with aging, macrophage-derived NLRP3 inflammasomes may cooperate with activated T cells to induce the development of inflammation in adipose tissue and the liver^107, 108^. Since TNF-α is an essential proinflammatory cytokine that drives the inflammatory process, whether dysfunctional adipose tissue induces systemic inflammation by secreting TNF-α in aging may need further investigation^109, 110^. Through the ERK/ETS1/interleukin-27Ra (IL27Ra) pathway, age-related TNF-α causes functional decline and myeloid bias of hematopoietic stem cells, which is related to myeloproliferative disease and immunosenescence^111^. Harmful products produced by dysregulated adipose tissue, such as lipids and free fatty acids, are also driving factors of inflammation^7^. Increased systemic free fatty acids, especially saturated fatty acids, promote the binding of monocytes to endothelial cells and proatherogenic cell surface antigen expression, which is a risk factor for atherosclerosis^112, 113, 114^.

**(4) Alterations in aging adipose tissue can lead to systematic changes by** **circulating miRNAs**

MiRNAs are small noncoding RNAs with 19–22 nucleotides that play critical roles in regulating mRNA metabolism. Circulating miRNAs are present in the extracellular environment, of which a large proportion are wrapped in exosomes^115^. In addition to being recognized as biomarker^116^s, miRNAs in systemic circulation could regulate the gene expression and function of distal cells by mediating paracrine and endocrine communication between different tissues. Adipose tissue has been demonstrated to be a crucial source of circulating miRNA, which may be involved in the communication between adipose tissue and other tissues. For instance, mouse miR-99b derived from BAT regulates FGF21 expression in the liver^117^. Decreased miRNA biogenesis due to the downregulation of Dicer and miRNA processing occurs at the level of the whole organism, including adipose tissue^117^. Interestingly, the beneficial effects of caloric restriction on the aging process rely on appropriate miRNAs from fat tissue, which may include circulating miRNAs^118^. To apply adipose-derived miRNAs for aging therapy, we still need more future studies to identify detailed miRNAs released from adipose tissue.

**(5) Obesity is a risk factor for age-related diseases**

It has long been established that obesity is one of the major risk factors for a range of age-related diseases, including diabetes, cancer, and cardiovascular disease^119^. There are many causes of obesity, including genetic mutation, physical inactivity and malnutrition, with the common result of abnormal accumulation of fat tissue. Obesity-related adipocyte hyperplasia in visceral white adipose tissue, which occurs more often in males than in females, may drive the age-associated redistribution of adipose tissue^120^. Obesity has been shown to accelerate the aging process in the liver, with nuclear mitochondrial genes involved in phosphorylation and electron transport^121^. In obesity, the excess presence of ROS due to fat accumulation increased Gfi1 expression in hematopoietic stem cells, which is associated with age-related hematological disorders ^122, 123, 124^. Increased retinol-binding protein-4 (RBP4) in obesity downregulates phosphatidylinositol-3-OH kinase (PI(3)K) signaling in muscle and promotes gluconeogenic enzyme phosphoenolpyruvate carboxykinase expression in the liver through a retinol-dependent mechanism, which induces insulin resistance^125^. For the cardiovascular disease, studies have revealed the significant association between obesity and elevated blood pressure, with obese individuals being 3.5 times more likely to develop hypertension^126^. Obesity-induced adipose tissue inflammation may be a driver of cancer risk, and certain adipokines (e.g., CCL2, VEGF, IL-6, and IL-8) act as chemotherapy inducers that enhance tumor cell migration and support metastasis. A recent study also found that obesity represses the infiltration and function of CD8+ T cells in the murine tumor microenvironment, which accelerates tumor growth^127^. Since obesity and the aging process share many pathogeneses and phenotypes, including impaired mitochondrial function, abnormal immunity, elevated systemic inflammation and insulin resistance, interventions for obesity could slow down the aging process.

**4. Adipose tissue as a therapeutic target in aging**

**(1) White adipose depots show early onset of aging**

Recently, a large-scale targeted proteomic analysis of a diverse panel of young versus old murine tissues revealed a significant aging effect on white adipose tissue, with alterations in lipid metabolism, central carbon metabolism, electron transport chain complexes, and inflammation ^128^. Another bulk RNA sequencing of 17 organs at 10 ages across the lifespan of mice suggested that WAT is an early onset of aging^3^. In WAT, a significant increase in differentially expressed genes in older mice compared to 3-month-old adults was detected at mid-age, which is earlier than that in other organs. Researchers analyzed the expression of genes involved in the age-associated immune response and found significant activation of cytokine-mediated inflammation in adipose tissue. Widespread activation of immune cells with accumulation of T cells and B cells, which is generally related to aging onset, was first detected in WAT during middle age^3^. By correlating plasma protein age trajectories with their corresponding gene expression trajectories in each organ, researchers also found that WAT is the source of several age-plasma proteins, which may accelerate the aging process throughout the body. In addition, impaired plasticity of subcutaneous WAT is already evident in middle-aged mice, which may be the early reason for insulin resistance^129^.

**(2) Systemic therapies extend lifespan by improving fat aging**

Strategies against the harmful influence of the aging process have recently been developed, some of which come into work by targeting adipose tissue. With the combination of dasatinib-a and quercetin-tyrosine kinase inhibitors (D+Q), Senolytics could alleviate physical dysfunction, including maximal walking speed, hanging endurance and grip strength, in parallel with extending lifespan and reducing mortality hazard in 24-27 months mice (equivalent to 75-90 years old human)^130^. In an open-label phase 1 pilot study of Senolytics, old patients with diabetic kidney disease treated with Senolytics showed a reduction in adipose tissue senescent cell burden and key SASP factors within 11 days^131^. In addition, a recent study demonstrated that aging-related alterations in the systemic environment partially originate in white adipose depots ^46^. Thus, intervention for adipose tissue aging may serve to repress age-related diseases and extend lifespan. In particular, the specific reduction in senescent cell accumulation and proinflammatory cytokine secretion in adipose tissue was the same as that in Senolysis-treated humans and mice, which supports that adipose tissue aging is a potential target for aging therapy^131^. Metformin is a well-known drug that has been used to treat T2D for more than 60 years and has been a popular star in anti-aging research in recent decades^132^. One of the mechanisms of Metformin in aging therapy is that Metformin improves PPAR and SREBP signaling, mitochondrial fatty acid oxidation, and collagen trimerization in adipose tissue^133^. A recent work reported that the low-dose PPARγ agonist thiazolidinedione (TZD) might be a novel pharmacological intervention to counteract aging and extend lifespan. Experiments in old mice showed that eWAT had the highest degree of gene expression changes in response to TZD treatment, specifically in inflammatory responses. Mice treated with TZD displayed improved age-dependent adipose tissue loss and reduced inflammation and fibrosis in aging WAT, contributing to the maintenance of adipose tissue homeostasis^134^. Heterochronic parabiosis studies with fusion of the circulatory systems of young mice and aged mice suggest that blood factors from younger donors can rejuvenate physiological function in old mice partially through diminished expression of cyclin-dependent kinase inhibitor (CDKi) genes p16 (Cdkn2a) and p21 (Cdkn1a/Cip1) in VAT^135^. To reach the beneficial effect of CR more efficiently, pharmacological approaches, named CR mimetics (CRMs), that mimic the role of CR in health have been introduced. Consistently, CRMs have profound effects on adipose tissue. Metformin, a popular CRM, prevents abnormal white adipocyte accumulation by increasing FGF21 expression^136^.

**(3) Adipose-specific interventions affect longevity**

Caloric restriction (CR) is a common strategy to prevent abnormal fat accumulation by chronic reduction of total calorie intake without malnutrition^137, 138^. Expression of genes associated with proliferator-activated receptor γ (PPARγ)-mediated adipogenesis lipid metabolism was downregulated with age but preserved by CR in WAT^139^. In addition to preventing obesity-related pathologies through weight loss, CR has also been broadly demonstrated to extend healthspan in most living organisms^140, 141^. CR treatment for 24 months had a beneficial effect in nonobese humans, which increased vigor, reduced mood disturbance and improved sleep quality^142^. An increasing underlying mechanism of CR against aging has been identified. As CR suppressed a substantial subset of the age-associated changes in WAT^139^, adipose tissue might act as an important mediator of the beneficial effects of CR, directly or indirectly. CR prevent the age-related accumulation of adipose tissue, which causes a series damage on adjacent or distance organs. Previous research suggested that sirtuin 1 (SIRT1) is the key molecular mediate the effect of CR on lifespan by inhibiting lipid accumulation and promoting lipolysis in adipocytes^143^. Moreover, CR with nutrient deprivation activates appropriate autophagy levels to remove dysfunctional organelles, proteins, and aggregates from the cytoplasm by regulating the expression of key genes, such as AMP-activated protein kinase (AMPK)^144^. The general reduction in mammalian target of rapamycin (mTOR) activity in aging can also be rescued to some degree by CR^139^. Interestingly, surgical removal of VAT in rats offered approximately 20% of the effect of CR on longevity with improving insulin action^145^.

Although highly controversial, gene therapy is still a promising treatment for various diseases and has moved from a vision to clinical reality. Finding the key gene target is fundamental for gene therapy against aging. Intriguingly, editing several adipose-related genes was shown to extend lifespan to varying degrees. The expression of *Nrip1* in visceral white adipose tissue (WAT) increases with aging, which might be associated with VAT expansion in aging. Nrip1 deletion in mice increases autophagy activity in periovarian white adipose tissue and reduces cellular senescence and proinflammatory cytokines in WAT, ultimately extending the health span^146^. Deleting Toll-like receptors in mice can alleviate inflammation at old age by reducing inflammation-related processes, including ER stress and senescence, which is a promising antiaging therapy^147^.

Together, adipose tissue interventions through lifestyle, drugs and gene editing can result in better health, suggesting that adipose tissue is a worthy target for treatment against aging.

**5. Summary**

As the lifespan of human beings has been extended greatly, it is important to find ways to reach healthful aging with physical and mental vigor. A large number of studies have emphasized the important role of adipose tissue in aging. Adipose tissue plays a crucial role in nutrient sensing, energy storage, and endocrine and immunological activity. Age-related adipose tissue alterations, including abnormal redistribution, decreased progenitor pool, accumulated senescent cells and activated inflammation, accelerate the aging process in the local environment, which can drive systemic adverse health outcomes with advancing age (Figure 4). Slowing down the aging process in adipose tissue is thought to prevent age-related disease. In this review, we provide mechanistic insights into the aging progression of adipose tissue with a cascade of molecular and cellular changes, as well as the underlying mechanism. Notably, some molecules derived from adipose tissue, such as free fatty acids, extracellular lipids and SASP, promote aging at the organismal level. Nevertheless, there remains some unresolved problem: What makes adipose tissue act at the onset of aging? Which cell type in adipose tissue is the origin of aging? Whether all adipocytes undergo aging synchronously? Is the role of the immune response in adipose aging a protector? Thanks to the rapid evolution of single-cell technologies, it has been possible to investigate the aging process of adipose tissue within several cell types. As adipose aging intervention has the potential to protect against systematic aging and age-related disease, more research is required to unveil the detailed mechanisms underlying fat aging and to provide a theoretical basis for antiaging therapy.

**Data availability**

The data used to support the findings of this study are available from the corresponding author upon request.

**References**

1. Coin A, Sergi G, Inelmen EM, Enzi G. *Pathophysiology of Body Composition Changes in Elderly People*. Cachexia and Wasting: A Modern Approach, 2006.

2. López-Otín C, Blasco MA, Partridge L, Serrano M, Kroemer G. The hallmarks of aging. *Cell* 2013, **153**(6)**:** 1194-1217.

3. Schaum N, Lehallier B, Hahn O, Palovics R, Hosseinzadeh S, Lee SE*, et al.* Ageing hallmarks exhibit organ-specific temporal signatures. *Nature* 2020, **583**(7817)**:** 596-602.

4. Schipper BM, Marra KG, Zhang W, Donnenberg AD, Rubin JP. Regional anatomic and age effects on cell function of human adipose-derived stem cells. *Annals of plastic surgery* 2008, **60**(5)**:** 538-544.

5. Park MH, Kim DH, Lee EK, Kim ND, Im DS, Lee J*, et al.* Age-related inflammation and insulin resistance: a review of their intricate interdependency. *Archives of pharmacal research* 2014, **37**(12)**:** 1507-1514.

6. Ghosh AK, Garg SK, Mau T, O'Brien M, Liu J, Yung R. Elevated Endoplasmic Reticulum Stress Response Contributes to Adipose Tissue Inflammation in Aging. *The journals of gerontology Series A, Biological sciences and medical sciences* 2015, **70**(11)**:** 1320-1329.

7. Camell CD, Sander J, Spadaro O, Lee A, Nguyen KY, Wing A*, et al.* Inflammasome-driven catecholamine catabolism in macrophages blunts lipolysis during ageing. *Nature* 2017, **550**(7674)**:** 119-123.

8. Rosen ED, Spiegelman BM. What we talk about when we talk about fat. *Cell* 2014, **156**(1-2)**:** 20-44.

9. Cinti S. Anatomy and physiology of the nutritional system. *Molecular aspects of medicine* 2019, **68:** 101-107.

10. Cannon B, Nedergaard J. Brown adipose tissue: function and physiological significance. *Physiol Rev* 2004, **84**(1)**:** 277-359.

11. Cinti S. The adipose organ at a glance. *Dis Model Mech* 2012, **5**(5)**:** 588-594.

12. Kuk JL, Saunders TJ, Davidson LE, Ross R. Age-related changes in total and regional fat distribution. *Ageing Res Rev* 2009, **8**(4)**:** 339-348.

13. Zuk PA, Zhu M, Ashjian P, De Ugarte DA, Huang JI, Mizuno H*, et al.* Human adipose tissue is a source of multipotent stem cells. *Mol Biol Cell* 2002, **13**(12)**:** 4279-4295.

14. Goldberg EL, Shchukina I, Youm YH, Ryu S, Tsusaka T, Young KC*, et al.* IL-33 causes thermogenic failure in aging by expanding dysfunctional adipose ILC2. *Cell metabolism* 2021.

15. Trim W, Turner JE, Thompson D. Parallels in Immunometabolic Adipose Tissue Dysfunction with Ageing and Obesity. *Frontiers in Immunology* 2018, **9**.

16. Starr ME, Hu Y, Stromberg AJ, Carmical JR, Wood TG, Evers BM*, et al.* Gene expression profile of mouse white adipose tissue during inflammatory stress: age-dependent upregulation of major procoagulant factors. *Aging cell* 2013, **12**(2)**:** 194-206.

17. Birch J, Gil J. Senescence and the SASP: many therapeutic avenues. *Genes Dev* 2020, **34**(23-24)**:** 1565-1576.

18. Scheja L, Heeren J. The endocrine function of adipose tissues in health and cardiometabolic disease. *Nat Rev Endocrinol* 2019, **15**(9)**:** 507-524.

19. Thompson D, Karpe F, Lafontan M, Frayn K. Physical activity and exercise in the regulation of human adipose tissue physiology. *Physiol Rev* 2012, **92**(1)**:** 157-191.

20. Hassan WU, Greiser U, Wang W. Role of adipose-derived stem cells in wound healing. *Wound Repair Regen* 2014, **22**(3)**:** 313-325.

21. Maeda K, Okubo K, Shimomura I, Mizuno K, Matsuzawa Y, Matsubara K. Analysis of an expression profile of genes in the human adipose tissue. *Gene* 1997, **190**(2)**:** 227-235.

22. Blüher M, Mantzoros CS. From leptin to other adipokines in health and disease: facts and expectations at the beginning of the 21st century. *Metabolism: clinical and experimental* 2015, **64**(1)**:** 131-145.

23. Ouchi N, Parker JL, Lugus JJ, Walsh K. Adipokines in inflammation and metabolic disease. *Nature reviews Immunology* 2011, **11**(2)**:** 85-97.

24. Santos AL, Sinha S. Obesity and aging: Molecular mechanisms and therapeutic approaches. *Ageing Res Rev* 2021, **67:** 101268.

25. Raguso CA, Kyle U, Kossovsky MP, Roynette C, Paoloni-Giacobino A, Hans D*, et al.* A 3-year longitudinal study on body composition changes in the elderly: role of physical exercise. *Clinical nutrition (Edinburgh, Scotland)* 2006, **25**(4)**:** 573-580.

26. Gavi S, Feiner JJ, Melendez MM, Mynarcik DC, Gelato MC, McNurlan MA. Limb fat to trunk fat ratio in elderly persons is a strong determinant of insulin resistance and adiponectin levels. *The journals of gerontology Series A, Biological sciences and medical sciences* 2007, **62**(9)**:** 997-1001.

27. Caso G, McNurlan MA, Mileva I, Zemlyak A, Mynarcik DC, Gelato MC. Peripheral fat loss and decline in adipogenesis in older humans. *Metabolism: clinical and experimental* 2013, **62**(3)**:** 337-340.

28. Lakowa N, Trieu N, Flehmig G, Lohmann T, Schön MR, Dietrich A*, et al.* Telomere length differences between subcutaneous and visceral adipose tissue in humans. *Biochem Biophys Res Commun* 2015, **457**(3)**:** 426-432.

29. Yoneshiro T, Aita S, Matsushita M, Kayahara T, Kameya T, Kawai Y*, et al.* Recruited brown adipose tissue as an antiobesity agent in humans. *J Clin Invest* 2013, **123**(8)**:** 3404-3408.

30. Berry DC, Jiang Y, Arpke RW, Close EL, Uchida A, Reading D*, et al.* Cellular Aging Contributes to Failure of Cold-Induced Beige Adipocyte Formation in Old Mice and Humans. *Cell metabolism* 2017, **25**(2)**:** 481.

31. Lee P, Swarbrick MM, Ho KK. Brown adipose tissue in adult humans: a metabolic renaissance. *Endocrine reviews* 2013, **34**(3)**:** 413-438.

32. Yoneshiro T, Ogawa T, Okamoto N, Matsushita M, Aita S, Kameya T*, et al.* Impact of UCP1 and β3AR gene polymorphisms on age-related changes in brown adipose tissue and adiposity in humans. *International journal of obesity (2005)* 2013, **37**(7)**:** 993-998.

33. Ma X, Xu L, Gavrilova O, Mueller E. Role of forkhead box protein A3 in age-associated metabolic decline. *Proc Natl Acad Sci U S A* 2014, **111**(39)**:** 14289-14294.

34. Vatner DE, Zhang J, Oydanich M, Guers J, Katsyuba E, Yan L*, et al.* Enhanced longevity and metabolism by brown adipose tissue with disruption of the regulator of G protein signaling 14. *Aging cell* 2018, **17**(4)**:** e12751.

35. Kirkland JL, Tchkonia T, Pirtskhalava T, Han J, Karagiannides I. Adipogenesis and aging: does aging make fat go MAD? *Experimental gerontology* 2002, **37**(6)**:** 757-767.

36. Zhu M, Kohan E, Bradley J, Hedrick M, Benhaim P, Zuk P. The effect of age on osteogenic, adipogenic and proliferative potential of female adipose-derived stem cells. *Journal of tissue engineering and regenerative medicine* 2009, **3**(4)**:** 290-301.

37. Kim SM, Lun M, Wang M, Senyo SE, Guillermier C, Patwari P*, et al.* Loss of white adipose hyperplastic potential is associated with enhanced susceptibility to insulin resistance. *Cell metabolism* 2014, **20**(6)**:** 1049-1058.

38. Guo W, Pirtskhalava T, Tchkonia T, Xie W, Thomou T, Han J*, et al.* Aging results in paradoxical susceptibility of fat cell progenitors to lipotoxicity. *American journal of physiology Endocrinology and metabolism* 2007, **292**(4)**:** E1041-1051.

39. Xu M, Palmer AK, Ding H, Weivoda MM, Pirtskhalava T, White TA*, et al.* Targeting senescent cells enhances adipogenesis and metabolic function in old age. *Elife* 2015, **4:** e12997.

40. Bellini E, Grieco MP, Raposio E. The science behind autologous fat grafting. *Annals of medicine and surgery (2012)* 2017, **24:** 65-73.

41. Hernandez-Segura A, Nehme J, Demaria M. Hallmarks of Cellular Senescence. *Trends in cell biology* 2018, **28**(6)**:** 436-453.

42. Schosserer M, Grillari J, Wolfrum C, Scheideler M. Age-Induced Changes in White, Brite, and Brown Adipose Depots: A Mini-Review. *Gerontology* 2018, **64**(3)**:** 229-236.

43. Tchkonia T, Morbeck DE, Von Zglinicki T, Van Deursen J, Lustgarten J, Scrable H*, et al.* Fat tissue, aging, and cellular senescence. *Aging cell* 2010, **9**(5)**:** 667-684.

44. de Magalhães JP, Passos JF. Stress, cell senescence and organismal ageing. *Mech Ageing Dev* 2018, **170:** 2-9.

45. Hall BM, Gleiberman AS, Strom E, Krasnov PA, Frescas D, Vujcic S*, et al.* Immune checkpoint protein VSIG4 as a biomarker of aging in murine adipose tissue. *Aging cell* 2020, **19**(10)**:** e13219.

46. Brigger D, Riether C, van Brummelen R, Mosher KI, Shiu A, Ding Z*, et al.* Eosinophils regulate adipose tissue inflammation and sustain physical and immunological fitness in old age. *Nat Metab* 2020, **2**(8)**:** 688-702.

47. Bapat SP, Myoung Suh J, Fang S, Liu S, Zhang Y, Cheng A*, et al.* Depletion of fat-resident Treg cells prevents age-associated insulin resistance. *Nature* 2015, **528**(7580)**:** 137-141.

48. Frasca D, Diaz A, Romero M, Landin AM, Blomberg BB. High TNF-α levels in resting B cells negatively correlate with their response. *Experimental gerontology* 2014, **54:** 116-122.

49. Salminen A. Activation of immunosuppressive network in the aging process. *Ageing Res Rev* 2020, **57:** 100998.

50. Sacks H, Symonds ME. Anatomical locations of human brown adipose tissue: functional relevance and implications in obesity and type 2 diabetes. *Diabetes* 2013, **62**(6)**:** 1783-1790.

51. Cedikova M, Kripnerová M, Dvorakova J, Pitule P, Grundmanova M, Babuska V*, et al.* Mitochondria in White, Brown, and Beige Adipocytes. *Stem cells international* 2016, **2016:** 6067349.

52. Bahler L, Verberne HJ, Admiraal WM, Stok WJ, Soeters MR, Hoekstra JB*, et al.* Differences in Sympathetic Nervous Stimulation of Brown Adipose Tissue Between the Young and Old, and the Lean and Obese. *Journal of nuclear medicine : official publication, Society of Nuclear Medicine* 2016, **57**(3)**:** 372-377.

53. Goto T, Naknukool S, Yoshitake R, Hanafusa Y, Tokiwa S, Li Y*, et al.* Proinflammatory cytokine interleukin-1β suppresses cold-induced thermogenesis in adipocytes. *Cytokine* 2016, **77:** 107-114.

54. Michaud M, Balardy L, Moulis G, Gaudin C, Peyrot C, Vellas B*, et al.* Proinflammatory cytokines, aging, and age-related diseases. *Journal of the American Medical Directors Association* 2013, **14**(12)**:** 877-882.

55. Soumano K, Desbiens S, Rabelo R, Bakopanos E, Camirand A, Silva JE. Glucocorticoids inhibit the transcriptional response of the uncoupling protein-1 gene to adrenergic stimulation in a brown adipose cell line. *Molecular and cellular endocrinology* 2000, **165**(1-2)**:** 7-15.

56. Doig CL, Fletcher RS, Morgan SA, McCabe EL, Larner DP, Tomlinson JW*, et al.* 11β-HSD1 Modulates the Set Point of Brown Adipose Tissue Response to Glucocorticoids in Male Mice. *Endocrinology* 2017, **158**(6)**:** 1964-1976.

57. Lin L, Saha PK, Ma X, Henshaw IO, Shao L, Chang BH*, et al.* Ablation of ghrelin receptor reduces adiposity and improves insulin sensitivity during aging by regulating fat metabolism in white and brown adipose tissues. *Aging cell* 2011, **10**(6)**:** 996-1010.

58. Qiang L, Wang L, Kon N, Zhao W, Lee S, Zhang Y*, et al.* Brown remodeling of white adipose tissue by SirT1-dependent deacetylation of Pparγ. *Cell* 2012, **150**(3)**:** 620-632.

59. Mota de Sá P, Richard AJ, Hang H, Stephens JM. Transcriptional Regulation of Adipogenesis. *Comprehensive Physiology* 2017, **7**(2)**:** 635-674.

60. Karagiannides I, Tchkonia T, Dobson DE, Steppan CM, Cummins P, Chan G*, et al.* Altered expression of C/EBP family members results in decreased adipogenesis with aging. *American journal of physiology Regulatory, integrative and comparative physiology* 2001, **280**(6)**:** R1772-1780.

61. Hotta K, Bodkin NL, Gustafson TA, Yoshioka S, Ortmeyer HK, Hansen BC. Age-related adipose tissue mRNA expression of ADD1/SREBP1, PPARgamma, lipoprotein lipase, and GLUT4 glucose transporter in rhesus monkeys. *The journals of gerontology Series A, Biological sciences and medical sciences* 1999, **54**(5)**:** B183-188.

62. Xu L, Ma X, Verma NK, Wang D, Gavrilova O, Proia RL*, et al.* Ablation of PPARγ in subcutaneous fat exacerbates age-associated obesity and metabolic decline. *Aging cell* 2018, **17**(2).

63. Karagiannides I, Thomou T, Tchkonia T, Pirtskhalava T, Kypreos KE, Cartwright A*, et al.* Increased CUG triplet repeat-binding protein-1 predisposes to impaired adipogenesis with aging. *J Biol Chem* 2006, **281**(32)**:** 23025-23033.

64. Tchkonia T, Pirtskhalava T, Thomou T, Cartwright MJ, Wise B, Karagiannides I*, et al.* Increased TNFalpha and CCAAT/enhancer-binding protein homologous protein with aging predispose preadipocytes to resist adipogenesis. *American journal of physiology Endocrinology and metabolism* 2007, **293**(6)**:** E1810-1819.

65. Fei J, Tamski H, Cook C, Santanam N. MicroRNA regulation of adipose derived stem cells in aging rats. *PLoS One* 2013, **8**(3)**:** e59238.

66. Di Micco R, Krizhanovsky V, Baker D, d'Adda di Fagagna F. Cellular senescence in ageing: from mechanisms to therapeutic opportunities. *Nat Rev Mol Cell Biol* 2021, **22**(2)**:** 75-95.

67. Calcinotto A, Kohli J, Zagato E, Pellegrini L, Demaria M, Alimonti A. Cellular Senescence: Aging, Cancer, and Injury. *Physiol Rev* 2019, **99**(2)**:** 1047-1078.

68. Gire V, Wynford-Thomas D. Reinitiation of DNA synthesis and cell division in senescent human fibroblasts by microinjection of anti-p53 antibodies. *Mol Cell Biol* 1998, **18**(3)**:** 1611-1621.

69. Baar MP, Brandt RMC, Putavet DA, Klein JDD, Derks KWJ, Bourgeois BRM*, et al.* Targeted Apoptosis of Senescent Cells Restores Tissue Homeostasis in Response to Chemotoxicity and Aging. *Cell* 2017, **169**(1)**:** 132-147.e116.

70. Freund A, Orjalo AV, Desprez PY, Campisi J. Inflammatory networks during cellular senescence: causes and consequences. *Trends Mol Med* 2010, **16**(5)**:** 238-246.

71. Baker DJ, Wijshake T, Tchkonia T, LeBrasseur NK, Childs BG, van de Sluis B*, et al.* Clearance of p16Ink4a-positive senescent cells delays ageing-associated disorders. *Nature* 2011, **479**(7372)**:** 232-236.

72. Kuilman T, Michaloglou C, Vredeveld LC, Douma S, van Doorn R, Desmet CJ*, et al.* Oncogene-induced senescence relayed by an interleukin-dependent inflammatory network. *Cell* 2008, **133**(6)**:** 1019-1031.

73. Krizhanovsky V, Yon M, Dickins RA, Hearn S, Simon J, Miething C*, et al.* Senescence of activated stellate cells limits liver fibrosis. *Cell* 2008, **134**(4)**:** 657-667.

74. Acosta JC, O'Loghlen A, Banito A, Guijarro MV, Augert A, Raguz S*, et al.* Chemokine signaling via the CXCR2 receptor reinforces senescence. *Cell* 2008, **133**(6)**:** 1006-1018.

75. Xu M, Tchkonia T, Ding H, Ogrodnik M, Lubbers ER, Pirtskhalava T*, et al.* JAK inhibition alleviates the cellular senescence-associated secretory phenotype and frailty in old age. *Proc Natl Acad Sci U S A* 2015, **112**(46)**:** E6301-6310.

76. Chen GY, Nuñez G. Sterile inflammation: sensing and reacting to damage. *Nature reviews Immunology* 2010, **10**(12)**:** 826-837.

77. Mack I, BelAiba RS, Djordjevic T, Görlach A, Hauner H, Bader BL. Functional analyses reveal the greater potency of preadipocytes compared with adipocytes as endothelial cell activator under normoxia, hypoxia, and TNFalpha exposure. *American journal of physiology Endocrinology and metabolism* 2009, **297**(3)**:** E735-748.

78. Starr ME, Evers BM, Saito H. Age-associated increase in cytokine production during systemic inflammation: adipose tissue as a major source of IL-6. *The journals of gerontology Series A, Biological sciences and medical sciences* 2009, **64**(7)**:** 723-730.

79. Jerschow E, Anwar S, Barzilai N, Rosenstreich D. Macrophages Accumulation in Visceral and Subcutaneous Adipose Tissue Correlates with Age. *Journal of Allergy & Clinical Immunology* 2007, **119**(1)**:** S179-S179.

80. Shook BA, Wasko RR, Mano O, Rutenberg-Schoenberg M, Rudolph MC, Zirak B*, et al.* Dermal Adipocyte Lipolysis and Myofibroblast Conversion Are Required for Efficient Skin Repair. *Cell Stem Cell* 2020, **26**(6)**:** 880-895 e886.

81. Ghosh AK, Mau T, O'Brien M, Garg S, Yung R. Impaired autophagy activity is linked to elevated ER-stress and inflammation in aging adipose tissue. *Aging* 2016, **8**(10)**:** 2525-2537.

82. Von Bank H, Kirsh C, Simcox J. Aging adipose: Depot location dictates age-associated expansion and dysfunction. *Ageing Res Rev* 2021, **67:** 101259.

83. Graja A, Garcia-Carrizo F, Jank AM, Gohlke S, Ambrosi TH, Jonas W*, et al.* Loss of periostin occurs in aging adipose tissue of mice and its genetic ablation impairs adipose tissue lipid metabolism. *Aging cell* 2018, **17**(5)**:** e12810.

84. Khan T, Muise ES, Iyengar P, Wang ZV, Chandalia M, Abate N*, et al.* Metabolic dysregulation and adipose tissue fibrosis: role of collagen VI. *Mol Cell Biol* 2009, **29**(6)**:** 1575-1591.

85. Minamino T, Orimo M, Shimizu I, Kunieda T, Yokoyama M, Ito T*, et al.* A crucial role for adipose tissue p53 in the regulation of insulin resistance. *Nat Med* 2009, **15**(9)**:** 1082-1087.

86. Serrano R, Villar M, Gallardo N, Carrascosa JM, Martinez C, Andrés A. The effect of aging on insulin signalling pathway is tissue dependent: central role of adipose tissue in the insulin resistance of aging. *Mech Ageing Dev* 2009, **130**(3)**:** 189-197.

87. Stienstra R, Joosten LA, Koenen T, van Tits B, van Diepen JA, van den Berg SA*, et al.* The inflammasome-mediated caspase-1 activation controls adipocyte differentiation and insulin sensitivity. *Cell metabolism* 2010, **12**(6)**:** 593-605.

88. Ballak DB, Stienstra R, Tack CJ, Dinarello CA, van Diepen JA. IL-1 family members in the pathogenesis and treatment of metabolic disease: Focus on adipose tissue inflammation and insulin resistance. *Cytokine* 2015, **75**(2)**:** 280-290.

89. Chondronikola M, Volpi E, Børsheim E, Porter C, Annamalai P, Enerbäck S*, et al.* Brown adipose tissue improves whole-body glucose homeostasis and insulin sensitivity in humans. *Diabetes* 2014, **63**(12)**:** 4089-4099.

90. Stanford KI, Lynes MD, Takahashi H, Baer LA, Arts PJ, May FJ*, et al.* 12,13-diHOME: An Exercise-Induced Lipokine that Increases Skeletal Muscle Fatty Acid Uptake. *Cell metabolism* 2018, **27**(5)**:** 1111-1120.e1113.

91. Lynes MD, Leiria LO, Lundh M, Bartelt A, Shamsi F, Huang TL*, et al.* The cold-induced lipokine 12,13-diHOME promotes fatty acid transport into brown adipose tissue. *Nat Med* 2017, **23**(5)**:** 631-637.

92. Zhu S, Tian Z, Torigoe D, Zhao J, Xie P, Sugizaki T*, et al.* Aging- and obesity-related peri-muscular adipose tissue accelerates muscle atrophy. *PLoS One* 2019, **14**(8)**:** e0221366.

93. Koehler EM, Schouten JN, Hansen BE, van Rooij FJ, Hofman A, Stricker BH*, et al.* Prevalence and risk factors of non-alcoholic fatty liver disease in the elderly: results from the Rotterdam study. *Journal of hepatology* 2012, **57**(6)**:** 1305-1311.

94. Item F, Konrad D. Visceral fat and metabolic inflammation: the portal theory revisited. *Obesity reviews : an official journal of the International Association for the Study of Obesity* 2012, **13 Suppl 2:** 30-39.

95. Fasshauer M, Blüher M. Adipokines in health and disease. *Trends in pharmacological sciences* 2015, **36**(7)**:** 461-470.

96. Münzberg H, Morrison CD. Structure, production and signaling of leptin. *Metabolism: clinical and experimental* 2015, **64**(1)**:** 13-23.

97. Scarpace PJ, Matheny M, Moore RL, Tümer N. Impaired leptin responsiveness in aged rats. *Diabetes* 2000, **49**(3)**:** 431-435.

98. Antuna-Puente B, Feve B, Fellahi S, Bastard JP. Adipokines: the missing link between insulin resistance and obesity. *Diabetes & metabolism* 2008, **34**(1)**:** 2-11.

99. Chitturi S, Farrell G, Frost L, Kriketos A, Lin R, Fung C*, et al.* Serum leptin in NASH correlates with hepatic steatosis but not fibrosis: a manifestation of lipotoxicity? *Hepatology (Baltimore, Md)* 2002, **36**(2)**:** 403-409.

100. McCullough AJ, Bugianesi E, Marchesini G, Kalhan SC. Gender-dependent alterations in serum leptin in alcoholic cirrhosis. *Gastroenterology* 1998, **115**(4)**:** 947-953.

101. Tsochatzis E, Papatheodoridis GV, Archimandritis AJ. The evolving role of leptin and adiponectin in chronic liver diseases. *The American journal of gastroenterology* 2006, **101**(11)**:** 2629-2640.

102. Yamauchi T, Kamon J, Waki H, Terauchi Y, Kubota N, Hara K*, et al.* The fat-derived hormone adiponectin reverses insulin resistance associated with both lipoatrophy and obesity. *Nat Med* 2001, **7**(8)**:** 941-946.

103. Waragai M, Ho G, Takamatsu Y, Shimizu Y, Sugino H, Sugama S*, et al.* Dual-therapy strategy for modification of adiponectin receptor signaling in aging-associated chronic diseases. *Drug discovery today* 2018, **23**(6)**:** 1305-1311.

104. Jamaluddin MS, Yan S, Lü J, Liang Z, Yao Q, Chen C. Resistin increases monolayer permeability of human coronary artery endothelial cells. *PLoS One* 2013, **8**(12)**:** e84576.

105. Fried SK, Bunkin DA, Greenberg AS. Omental and subcutaneous adipose tissues of obese subjects release interleukin-6: depot difference and regulation by glucocorticoid. *The Journal of clinical endocrinology and metabolism* 1998, **83**(3)**:** 847-850.

106. Fain JN, Madan AK, Hiler ML, Cheema P, Bahouth SW. Comparison of the release of adipokines by adipose tissue, adipose tissue matrix, and adipocytes from visceral and subcutaneous abdominal adipose tissues of obese humans. *Endocrinology* 2004, **145**(5)**:** 2273-2282.

107. Vandanmagsar B, Youm YH, Ravussin A, Galgani JE, Stadler K, Mynatt RL*, et al.* The NLRP3 inflammasome instigates obesity-induced inflammation and insulin resistance. *Nat Med* 2011, **17**(2)**:** 179-188.

108. Ortega Martinez de Victoria E, Xu X, Koska J, Francisco AM, Scalise M, Ferrante AW, Jr.*, et al.* Macrophage content in subcutaneous adipose tissue: associations with adiposity, age, inflammatory markers, and whole-body insulin action in healthy Pima Indians. *Diabetes* 2009, **58**(2)**:** 385-393.

109. Morin CL, Gayles EC, Podolin DA, Wei Y, Xu M, Pagliassotti MJ. Adipose tissue-derived tumor necrosis factor activity correlates with fat cell size but not insulin action in aging rats. *Endocrinology* 1998, **139**(12)**:** 4998-5005.

110. Nosalski R, Guzik TJ. Perivascular adipose tissue inflammation in vascular disease. *British journal of pharmacology* 2017, **174**(20)**:** 3496-3513.

111. He H, Xu P, Zhang X, Liao M, Dong Q, Cong T*, et al.* Aging-induced IL27Ra signaling impairs hematopoietic stem cells. *Blood* 2020, **136**(2)**:** 183-198.

112. Mata P, Alonso R, Lopez-Farre A, Ordovas JM, Lahoz C, Garces C*, et al.* Effect of dietary fat saturation on LDL oxidation and monocyte adhesion to human endothelial cells in vitro. *Arteriosclerosis, thrombosis, and vascular biology* 1996, **16**(11)**:** 1347-1355.

113. Zhang WY, Schwartz E, Wang Y, Attrep J, Li Z, Reaven P. Elevated concentrations of nonesterified fatty acids increase monocyte expression of CD11b and adhesion to endothelial cells. *Arteriosclerosis, thrombosis, and vascular biology* 2006, **26**(3)**:** 514-519.

114. Ghosh A, Gao L, Thakur A, Siu PM, Lai CWK. Role of free fatty acids in endothelial dysfunction. *Journal of biomedical science* 2017, **24**(1)**:** 50.

115. Arroyo JD, Chevillet JR, Kroh EM, Ruf IK, Pritchard CC, Gibson DF*, et al.* Argonaute2 complexes carry a population of circulating microRNAs independent of vesicles in human plasma. *Proc Natl Acad Sci U S A* 2011, **108**(12)**:** 5003-5008.

116. Chen X, Ba Y, Ma L, Cai X, Yin Y, Wang K*, et al.* Characterization of microRNAs in serum: a novel class of biomarkers for diagnosis of cancer and other diseases. *Cell Res* 2008, **18**(10)**:** 997-1006.

117. Thomou T, Mori MA, Dreyfuss JM, Konishi M, Sakaguchi M, Wolfrum C*, et al.* Adipose-derived circulating miRNAs regulate gene expression in other tissues. *Nature* 2017, **542**(7642)**:** 450-455.

118. Reis FC, Branquinho JL, Brandão BB, Guerra BA, Silva ID, Frontini A*, et al.* Fat-specific Dicer deficiency accelerates aging and mitigates several effects of dietary restriction in mice. *Aging* 2016, **8**(6)**:** 1201-1222.

119. Burton DGA, Faragher RGA. Obesity and type-2 diabetes as inducers of premature cellular senescence and ageing. *Biogerontology* 2018, **19**(6)**:** 447-459.

120. Jeffery E, Wing A, Holtrup B, Sebo Z, Kaplan JL, Saavedra-Peña R*, et al.* The Adipose Tissue Microenvironment Regulates Depot-Specific Adipogenesis in Obesity. *Cell metabolism* 2016, **24**(1)**:** 142-150.

121. Horvath S, Erhart W, Brosch M, Ammerpohl O, von Schönfels W, Ahrens M*, et al.* Obesity accelerates epigenetic aging of human liver. *Proc Natl Acad Sci U S A* 2014, **111**(43)**:** 15538-15543.

122. Furukawa S, Fujita T, Shimabukuro M, Iwaki M, Yamada Y, Nakajima Y*, et al.* Increased oxidative stress in obesity and its impact on metabolic syndrome. *J Clin Invest* 2004, **114**(12)**:** 1752-1761.

123. Lee JM, Govindarajah V, Goddard B, Hinge A, Muench DE, Filippi MD*, et al.* Obesity alters the long-term fitness of the hematopoietic stem cell compartment through modulation of Gfi1 expression. *The Journal of experimental medicine* 2018, **215**(2)**:** 627-644.

124. Akunuru S, Geiger H. Aging, Clonality, and Rejuvenation of Hematopoietic Stem Cells. *Trends Mol Med* 2016, **22**(8)**:** 701-712.

125. Yang Q, Graham TE, Mody N, Preitner F, Peroni OD, Zabolotny JM*, et al.* Serum retinol binding protein 4 contributes to insulin resistance in obesity and type 2 diabetes. *Nature* 2005, **436**(7049)**:** 356-362.

126. Seravalle G, Grassi G. Obesity and hypertension. *Pharmacological research* 2017, **122:** 1-7.

127. Ringel AE, Drijvers JM, Baker GJ, Catozzi A, Garcia-Canaveras JC, Gassaway BM*, et al.* Obesity Shapes Metabolism in the Tumor Microenvironment to Suppress Anti-Tumor Immunity. *Cell* 2020, **183**(7)**:** 1848-1866 e1826.

128. Yu Q, Xiao H, Jedrychowski MP, Schweppe DK, Navarrete-Perea J, Knott J*, et al.* Sample multiplexing for targeted pathway proteomics in aging mice. *Proc Natl Acad Sci U S A* 2020, **117**(18)**:** 9723-9732.

129. Corrales P, Vivas Y, Izquierdo-Lahuerta A, Horrillo D, Seoane-Collazo P, Velasco I*, et al.* Long-term caloric restriction ameliorates deleterious effects of aging on white and brown adipose tissue plasticity. *Aging cell* 2019, **18**(3)**:** e12948.

130. Xu M, Pirtskhalava T, Farr JN, Weigand BM, Palmer AK, Weivoda MM*, et al.* Senolytics improve physical function and increase lifespan in old age. *Nat Med* 2018, **24**(8)**:** 1246-1256.

131. Hickson LJ, Langhi Prata LGP, Bobart SA, Evans TK, Giorgadze N, Hashmi SK*, et al.* Senolytics decrease senescent cells in humans: Preliminary report from a clinical trial of Dasatinib plus Quercetin in individuals with diabetic kidney disease. *EBioMedicine* 2019, **47:** 446-456.

132. Soukas AA, Hao H, Wu L. Metformin as Anti-Aging Therapy: Is It for Everyone? *Trends Endocrinol Metab* 2019, **30**(10)**:** 745-755.

133. Kulkarni AS, Brutsaert EF, Anghel V, Zhang K, Bloomgarden N, Pollak M*, et al.* Metformin regulates metabolic and nonmetabolic pathways in skeletal muscle and subcutaneous adipose tissues of older adults. *Aging cell* 2018, **17**(2).

134. Xu L, Ma X, Verma N, Perie L, Pendse J, Shamloo S*, et al.* PPARγ agonists delay age-associated metabolic disease and extend longevity. *Aging cell* 2020, **19**(11)**:** e13267.

135. Ghosh AK, O'Brien M, Mau T, Qi N, Yung R. Adipose Tissue Senescence and Inflammation in Aging is Reversed by the Young Milieu. *The journals of gerontology Series A, Biological sciences and medical sciences* 2019, **74**(11)**:** 1709-1715.

136. Kim EK, Lee SH, Jhun JY, Byun JK, Jeong JH, Lee SY*, et al.* Metformin Prevents Fatty Liver and Improves Balance of White/Brown Adipose in an Obesity Mouse Model by Inducing FGF21. *Mediators of inflammation* 2016, **2016:** 5813030.

137. Das M, Gabriely I, Barzilai N. Caloric restriction, body fat and ageing in experimental models. *Obesity reviews : an official journal of the International Association for the Study of Obesity* 2004, **5**(1)**:** 13-19.

138. Barzilai N, Gupta G. Revisiting the role of fat mass in the life extension induced by caloric restriction. *The journals of gerontology Series A, Biological sciences and medical sciences* 1999, **54**(3)**:** B89-96; discussion B97-88.

139. Linford NJ, Beyer RP, Gollahon K, Krajcik RA, Malloy VL, Demas V*, et al.* Transcriptional response to aging and caloric restriction in heart and adipose tissue. *Aging cell* 2007, **6**(5)**:** 673-688.

140. Madeo F, Carmona-Gutierrez D, Hofer SJ, Kroemer G. Caloric Restriction Mimetics against Age-Associated Disease: Targets, Mechanisms, and Therapeutic Potential. *Cell metabolism* 2019, **29**(3)**:** 592-610.

141. Masoro EJ. Caloric restriction and aging: controversial issues. *The journals of gerontology Series A, Biological sciences and medical sciences* 2006, **61**(1)**:** 14-19.

142. Martin CK, Bhapkar M, Pittas AG, Pieper CF, Das SK, Williamson DA*, et al.* Effect of Calorie Restriction on Mood, Quality of Life, Sleep, and Sexual Function in Healthy Nonobese Adults: The CALERIE 2 Randomized Clinical Trial. *JAMA internal medicine* 2016, **176**(6)**:** 743-752.

143. Picard F, Guarente L. Molecular links between aging and adipose tissue. *International journal of obesity (2005)* 2005, **29 Suppl 1:** S36-39.

144. Cantó C, Auwerx J. Calorie restriction: is AMPK a key sensor and effector? *Physiology (Bethesda, Md)* 2011, **26**(4)**:** 214-224.

145. Muzumdar R, Allison DB, Huffman DM, Ma X, Atzmon G, Einstein FH*, et al.* Visceral adipose tissue modulates mammalian longevity. *Aging cell* 2008, **7**(3)**:** 438-440.

146. Wang J, Chen X, Osland J, Gerber SJ, Luan C, Delfino K*, et al.* Deletion of Nrip1 Extends Female Mice Longevity, Increases Autophagy, and Delays Cell Senescence. *The journals of gerontology Series A, Biological sciences and medical sciences* 2018, **73**(7)**:** 882-892.

147. Ghosh AK, O'Brien M, Mau T, Yung R. Toll-like receptor 4 (TLR4) deficient mice are protected from adipose tissue inflammation in aging. *Aging* 2017, **9**(9)**:** 1971-1982.

**Acknowledgements**

Figures were modified from the BioRender templates (https://app.biorender.com/).

**Funding**

This work was supported by the National Natural Science Foundation of China (81620108019, 81971848), Clinical Research Plan of SHDC (SHDC2020CR1019B, SHDC2020CR4029), Shanghai Municipal Key Clinical Specialty (shslczdzk00901), and Innovative Research Team of High-level Local University in Shanghai (SSMU-ZDCX20180700).

**Author information**

**Affiliations**

**Department of Plastic & Reconstructive Surgery, Shanghai Ninth People’s Hospital, Shanghai Jiao Tong University School of Medicine, Shanghai, China**

Min-Yi Ou, Hao Zhang, Poh-Ching Tan, Shuang-Bai Zhou, Qing-Feng Li

**Contributions**

M.Y.O, H.Z. and P.C.T. researched the data for the article and drafted the manuscript. S.B.Z. and Q.F.L. conceived the manuscript.

**Corresponding authors**

Correspondence to Shuangbai Zhou or Qingfeng Li.

**Ethics declarations**

Competing interests

The authors declared no competing interests.

**Figure legend**

**Figure 1.** **The age-related redistribution of adipose tissue.**

There is a redistribution of WAT mass with advancing age, displayed by increased visceral fat and reduced subcutaneous fat. Yellow represents subcutaneous fat, and red represents visceral fat.

**Figure 2. Age-related alteration in adipose tissue with dysregulated immune cells, preadipocytes and senescent cells.**

In young WAT, APSCs actively proliferate and differentiate to adipocyte. Resident immune cells keep in a relatively stable state. With advancing aging, APSCs gradually loss the developmental potential, leading to low adipogenesis. Aberrant immune cells and senescent cells accumulation drive the aging process of adipose. This figure was created in part with modified BioRender templates.

**Figure 3. Mechanisms of impaired adipogenesis with aging.**

In the aging process, the expression of pro-adipogenic genes, such as C/EBPα and PPARγ, declines in adipose progenitor and stem cells (APSCs), accompanied by increased levels of antiadipogenic factors, including C/EBPβ-LIP, CHOP and CUGBP1. As a result, old APSCs show a decline in proliferation and differentiation, which leads to low adipogenesis in the elderly. This figure was created in part with modified BioRender templates.

**Figure 4 The role of adipose tissue in aging process.**

In aging individuals, the adipose tissue can be characterized by tissue redistribution, reduced brown and beige fat, declined APSCs, senescent cells accumulation with SASP and dysregulated immune cells. Aging adipose impacts the elderly with ectopic lipid and FFA, dysregulated adiponectin, increased proinflammatory cytokines, reduced miRNA synthesis and high ROS activity. This figure was created in part with modified BioRender templates.
